# Combined surgery and chondrocyte cell-sheet transplantation improves clinical and structural outcomes in knee osteoarthritis

**DOI:** 10.1038/s41536-019-0069-4

**Published:** 2019-02-21

**Authors:** Masato Sato, Masayuki Yamato, Genya Mitani, Tomonori Takagaki, Kosuke Hamahashi, Yoshihiko Nakamura, Miya Ishihara, Ryo Matoba, Hiroyuki Kobayashi, Teruo Okano, Joji Mochida, Masahiko Watanabe

**Affiliations:** 1 Department of Orthopaedic Surgery, Surgical Science, Tokai University School of Medicine, 143 Shimokasuya, Isehara, Kanagawa 259-1193 Japan; 2 Institute of Advanced Biomedical Engineering and Science, Tokyo Women’s Medical University, 8-1 Kawada-cho, Shinjuku, Tokyo, 162-8666 Japan; 3 Cell Processing Center, Tokai University Hospital, 143 Shimokasuya, Isehara, Kanagawa 259-1193 Japan; 4 Department of Medical Engineering, National Defense Medical College, 3-2 Namiki, Tokorozawa, Saitama, 359-8513 Japan; 5 DNA Chip Research Inc., 1-15-1 Kaigan, Suzue Baydium 5F Minato-ku, Tokyo, 105-0022 Japan; 6 Department of Clinical Pharmacology, Tokai University School of Medicine, 143 Shimokasuya, Isehara, Kanagawa 259-1193 Japan

## Abstract

Current cartilage regenerative therapies are not fully effective in treating osteoarthritis of the knee (OAK). We have developed chondrocyte sheets for autologous transplantation and tested these in in vitro and in vivo preclinical studies, and have reported that the transplantation of chondrocyte sheets promoted hyaline cartilage repair in rat, rabbit, and minipig models. However, autologous transplantation of chondrocyte sheets has yet to be reported in humans. Here, we report our combination therapy in which conventional surgical treatment for OAK, is followed by autologous chondrocyte sheet transplantation for cartilage repair. Eight patients with OAK and cartilage defects categorized arthroscopically as Outerbridge grade III or IV receive the therapy. Patients are thoroughly assessed by preoperative and postoperative X-rays, magnetic resonance imaging (MRI), arthroscopy, Knee injury and Osteoarthritis Outcome Score (KOOS), Lysholm Knee Score (LKS), and a laser-induced photoacoustic method to assess cartilage viscoelasticity. Arthroscopic biopsies of all patients are performed 12 months after transplantation for histological evaluation. The properties of the chondrocyte sheets are evaluated using gene expression analysis to investigate the ability to predict the clinical and structural outcomes of the therapy. For this small initial longitudinal series, combination therapy is effective, as assessed by MRI, arthroscopy, viscoelasticity, histology, and the clinical outcomes of KOOS and LKS. Gene marker sets identified in autologous chondrocyte sheets may be predictive of the overall KOOS, LKS, and histological scores after therapy. These predictive gene sets may be potential alternative markers for evaluating OAK treatment.

## Osteoarthritis: Cartilage sheets help repair knee damage

Cell-sheet technology combined with conventional surgery offers a promising new treatment for osteoarthritis of the knee, according to a small clinical study. Masato Sato and colleagues from the Tokai University School of  Medicine in Isehara, Japan, collected cartilage cells from eight patients with degenerative, wear-and-tear arthritis of the knee. They fashioned the cells into layered sheets, and then transplanted these tissues back into the patients’ knees following surgical reconstruction of the joint or ligament. Diagnostic imaging, clinical evaluations and functional tests showed that the combination therapy was effective. One year post-transplant, the researchers then took tiny biopsies of the cartilage tissue and examined the regenerated cartilage. Gene expression analysis of cell sheets revealed several genes linked to favorable clinical and structural outcomes that could serve as predictive biomarkers following this novel regenerative treatment strategy.

## Introduction

Articular cartilage is a matrix-rich, hypocellular, and vasculature-free structure that functions as a lubricating and load-bearing surface in the joints. Injury to cartilage often progresses spatiotemporally from the articular surface to the subchondral bone and leads to the development of degenerative joint diseases such as osteoarthritis (OA).^1^ OA is the most common cause of mobility loss that adversely affects quality of life, work productivity, and cost of health care, and is the most prevalent form of musculoskeletal disease worldwide.^2,3^ No cure or proven interventions are known to stop OA progression. Since 1997, four representative autologous chondrocyte implantation products have been approved in the United States, the European Union, and Japan.^4^ However, the adaptation of such therapies to cartilaginous defects caused by osteoarthritis of the knee (OAK) might prove difficult because cartilage defects in OAK are variable and changes are associated with female sex, age, and body mass index. Increases of defects are associated with baseline cartilage volume, bone size, and osteophytes, which suggests that these factors play a role in the pathogenesis of cartilage defects.^5^

OA is a heterogeneous disease characterized by variable clinical features, biochemical and genetic characteristics, and responses to treatments.^6^ If a control group is used for a comparative study, it always includes individuals with heterogeneous conditions. OAK-related cartilage defects develop over many years and often require multiple therapies to treat coexisting pathological conditions such as malalignment and ligamentous and meniscal disorders. Therefore, complying with regulations that require clear evidence of the add-on effect of each treatment makes the development of new therapies particularly challenging.

Cell-sheet technology has been successfully implemented in clinical research for the regeneration of tissues such as the cornea,^7^ myocardium,^8^ and esophagus.^9^ We were the first to report its applicability in articular cartilage repair with the development of layered chondrocyte sheets.^10,11^ We have provided evidence from animal studies indicating the potential of layered chondrocyte sheets in the treatment of a partial defect in rabbit cartilage,^10^ and osteochondral defects in rat,^12^ rabbit,^13,14^ and minipig^15^ cartilage. These are the types of defects that are usually present in OAK. We have also investigated the mode of action of the layered chondrocyte sheets.^11,16,17^ To confirm the safety of the sheets, we have performed tumorigenicity studies as recommended by the World Health Organization,^18,19^ monitored genetic mutations and the emergence of abnormal chromosomes,^19^ and transplanted luciferase-expressing chondrocyte sheets and followed them for over 21 months to track their intra-articular localization.^12^ On the basis of these data, we received approval from the Ministry of Health, Labor and Welfare of Japan to conduct a clinical study targeting OAK.^20^

**Fig. 1:**
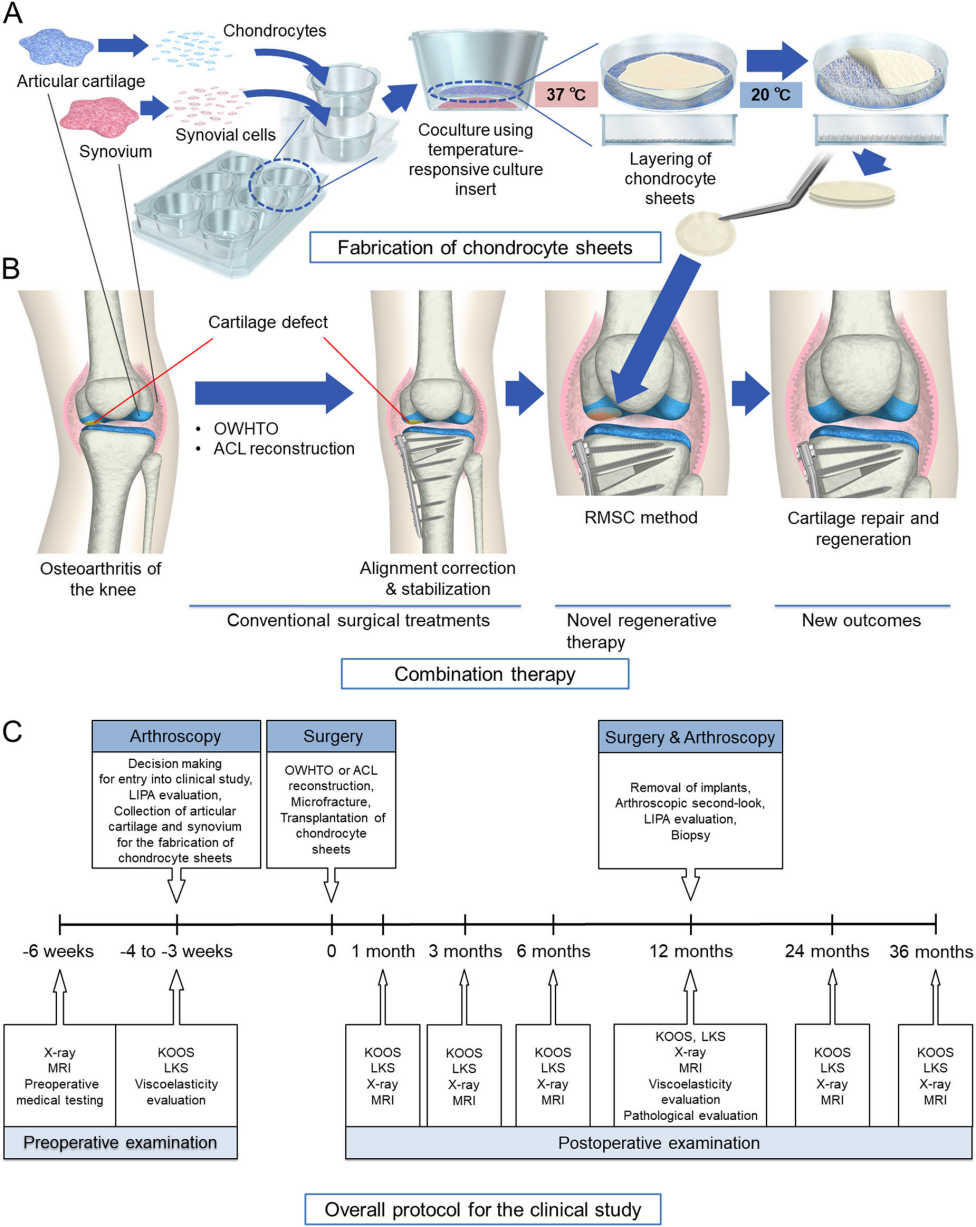
Treatments using a combination of conventional surgical interventions and transplantation of chondrocyte sheets were performed. **a** Chondrocyte sheets were fabricated from autologous articular cartilage (1.5 g) obtained from nonloading regions of the knee and synovium (3 g) collected during arthroscopy. In the cell-processing center, isolated chondrocytes and synovial cells were cocultured for 14–17 days using temperature-responsive culture inserts and were subsequently layered by detaching each chondrocyte sheet by changing the temperature from 37 to 20 °C. The layered chondrocyte sheets were cultured for another 7–8 days before transplantation. **b** OAK patients were first treated with conventional surgical treatments OWHTO or ACL reconstruction, followed by the removal of unhealthy tissue, marrow stimulation, and chondrocyte sheet transplantation (RMSC method). **c** The overall protocol for the clinical study meant that the final decision about entry into the clinical study was made during arthroscopic evaluation. For patients meeting the inclusion criteria, LIPA was used to assess their cartilage defects, and cartilage and synovial tissues were collected for the fabrication of chondrocyte sheets

We wanted to challenge the nature of the current regulations by applying cell-sheet technology^21,22^ combined with conventional surgical treatments and rigorous follow-up procedures in the first human clinical study of regenerative medicine applied to OAK in Japan. We designed a combination therapy (Fig. 1a, b) in which conventional surgical treatment for OAK, covered by National Health Insurance, was followed by the removal of unhealthy tissue, marrow stimulation, and chondrocyte sheet transplantation (the RMSC method) to treat cartilage defects. The study was designed to evaluate the effectiveness of the total treatment but not the add-on effects of each treatment.

Ten patients with OAK were enrolled, and eight patients with accompanying cartilage defects received the therapy. A rigorous evaluation protocol (Fig. 1c) was designed to assess the endpoints of safety and efficacy of the therapy and to evaluate the clinical and structural outcomes. The properties of the transplanted chondrocyte sheets were also evaluated thoroughly using gene expression analysis to investigate their potential use for predicting the clinical and structural outcomes of the therapy. In this small initial longitudinal case series, we wanted to determine whether expression of specific marker gene sets in autologous transplanted chondrocyte sheets derived from each patient could be used to predict the outcomes of this new therapy for OAK.

## Results

### Clinical outcomes of the combination therapy using RMSC method

**Fig. 2:**
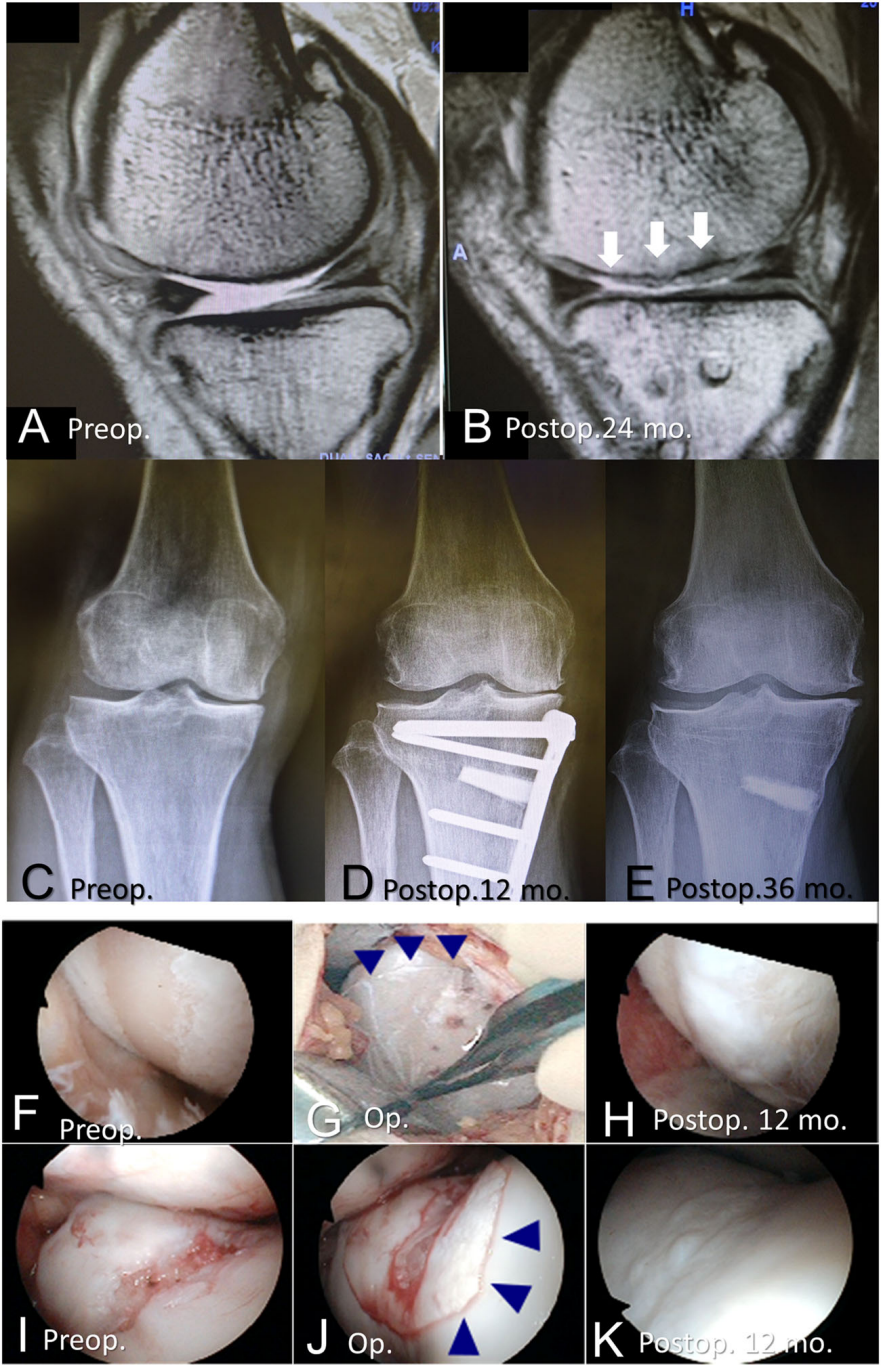
Representative magnetic resonance imaging (**a**, **b**), X-ray photographs (**c**–**e**), and arthroscopic images (**f**–**k**). **a**–**e** (patient 8), **f**–**h** (patient 6), and **i**–**k** (patient 2). **a**, **b** T2-weighted images were obtained using a 3.0-Tesla instrument. **a** Lack of articular cartilage and accumulation of synovial fluid were observed preoperatively. **b** Regeneration of articular cartilage, indicated by the arrows, was confirmed 24 months postoperatively. **c** X-ray photographs confirmed a preoperative varus deformity and narrowing of the medial joint space. **d** X-ray photographs 12 months postoperatively showed that the alignment was maintained after OWHTO. **e** At 36 months postoperatively (24 months after implants were removed), the alignment was maintained and the β-tricalcium phosphate graft had been absorbed. The medial osteophyte progression was observed in this patient. **f**–**h** The cartilage defect of the medial femoral condyle caused exposure of the subchondral bone. **i**–**k** The cartilage defect in the trochlear groove of the femur is shown. **f**, **i** Preoperative cartilage defects are shown. **g**, **j** Chondrocyte sheets were transplanted. **h**, **k** The presence of regenerated cartilage was confirmed at 12 months postoperatively

The results of clinical examinations and outcomes for the eight patients who received the combination therapy are shown in Table 1. Data from three of eight patients who underwent the therapy are shown in Fig. 2. No serious adverse events related to the combined surgeries were observed during the treatment and follow-up period. There were 26 adverse events reported, including pain (eight patients), leukocytosis (eight patients), increased C-reactive protein level (eight patients), swelling (one patient), and fever (one patient). All adverse events were considered to have been related to the high tibial osteotomy or anterior cruciate ligament reconstruction concomitant with the transplantation of chondrocyte sheets. We believe that these were unlikely to have been related to the transplantation of chondrocyte sheets.

**Table 1:** Clinical examinations and outcomes

Patient number	1	2	3	4	5	6	7	8
Age (years)	42	45	30	50	52	59	55	54
BMI (kg/m^2^)	29.9	25.5	27.0	24.0	20.7	30.6	22.7	21.2
Duration of symptoms (years)	20	15	8	5	9	12	15	10
Type of OA	PT	PT	PT	Gen	Gen	Gen	Gen	Gen
Location of defect	LFC	LFC, PF	MFC	MFC	MFC	MFC	MFC	MFC, PF
Size of defect (cm^2^)	3.38	2.25 3.20	3.92	4.10	3.98	3.12	4.02	3.57 2.88
Outerbridge classification	3	3	3	4	4	4	4	4
KL grade	2	2	2	3	4	3	3	3
Surgical treatments	ACLR	ACLR	ACLR	OWHTO	OWHTO	OWHTO	OWHTO	OWHTO
Thickness of RC (mm)	3.5	LFC 2.4 PF 4.2	3.1	3.2	3.6	3.4	2.4	MFC 2.1 PF 2.9
Viscoelasticity of RC by LIPA (preop. value)	0.87 (0.65)	LFC 1.04 (0.74) PF 1.06 (0.60)	0.98 (0.78)	0.87 (0.52)	0.89 (0.70)	0.90 (0.60)	0.88 (0.69)	MFC 0.92 (0.58) PF 0.88 (ND)
OARSI histological score	4	9	2	12	3	3	5	14
Mankin score	3	7	4	8	3	4	3	6
Histological appearance	HL	HL	HL	HL	HL	HL	HL	HL
Last follow-up date (months)	61	49	36	47	36	36	36	36

*BMI* body mass index, *OA* osteoarthritis, *PT* post-traumatic, *Gen* general, *LFC* lateral femoral condyle, *PF* patellofemoral groove, *MFC* medial femoral condyle, *KL grade* Kellgren–Lawrence grade, *ACLR* anterior cruciate ligament reconstruction, *OWHTO* open-wedge high tibial osteotomy, *RC* regenerated cartilage, *LIPA* laser-induced photoacoustic measurement, *ND* not detectable, *OARSI* Osteoarthritis Research Society International, *HL* hyaline-like cartilage

**Fig. 3:**
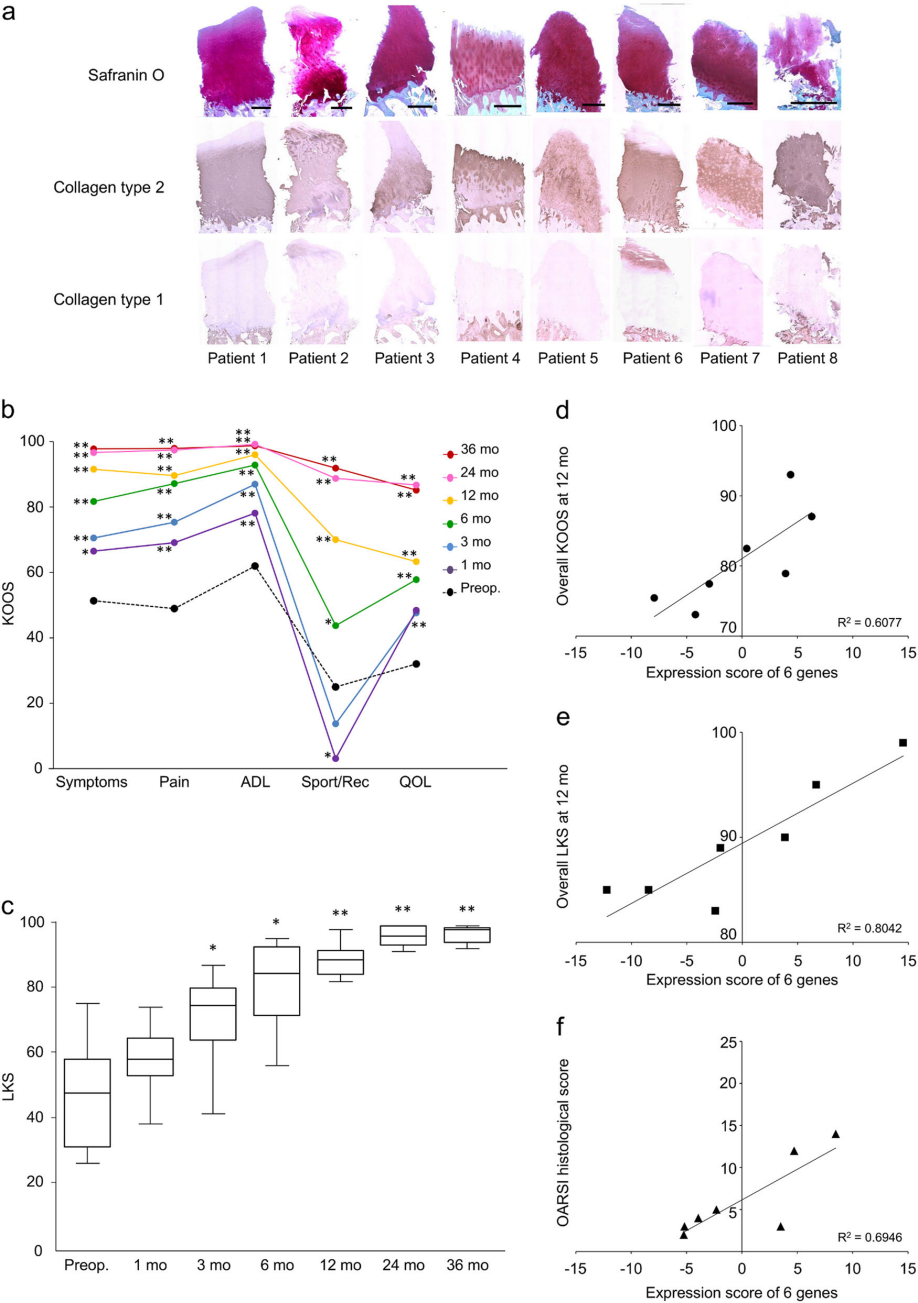
Structural and clinical results. **a** Histological results of patient biopsies and results from clinical evaluations are shown. Biopsies were taken 12 months postoperatively from areas of regenerated cartilage. Histological sections of samples from all patients stained strongly for Safranin O; scale bar = 500 μm. Immunostaining showed strong expression of type II collagen (COL2) in all patients and weak expressions of type I collagen (COL1) near the articular cartilage surface in patients 1, 2, and 6. **b** KOOS was used to assess the effects on knee-related parameters pre- and postoperatively. The patient averages for all KOOS subscores improved significantly from the baseline and indicated recovery of function in sport and recreation (Sport/Rec) after the patients were permitted to return to such activities. *p < 0.05, **p < 0.01. ADL, activities of daily living; QOL, quality of life. **c** Patient averages for LKS improved significantly from the baseline. *p < 0.05, **p < 0.01. **d** Correlational analysis was used to select a gene marker set predictive of overall KOOS at 12 months postoperatively (*ACAN*, *CSGALNACT1*, *BMP6*, *PTGS2*, *POU5F1*, and *CXCL6*); **e** LKS at 12 months postoperatively (*GATA6*, *LECT1*, *GDF5*, *ADAMTS5*, *SP7*, and *MATN2*); and **f** OARSI histological score (*ACAN*, *BMP6*, *POU5F1*, *CXCL6*, *VEGFA*, and *PTGS2*)

Scores for the individual subscales of the Knee injury and Osteoarthritis Outcome Score (KOOS) continued to improve up to 36 months postoperatively (Fig. 3b). The Lysholm Knee Score (LKS) continued to improve up to 24 months postoperatively and was maintained thereafter (Fig. 3c).

### Structural outcomes of the regenerated cartilage

At 36 months postoperatively, MRI confirmed the regeneration of cartilage in areas that lacked cartilage preoperatively (Fig. 2a, b). The results of the magnetic resonance observation of cartilage repair tissue (MOCART) evaluation are shown in Table 2. Knee alignment was maintained 36 months postoperatively (Fig. 2c–e). Cartilage regeneration in the defect areas was confirmed by preoperative, intratransplantation, and postoperative arthroscopy (Fig. 2f–k).

**Table 2:** MRI evaluation of repair tissue 3 years after the combination therapy, by number and percentage

Variables	Number (percentage)
1. Degree of defect repair and filling of the defect	
Complete	5 (62.5)
Hypertrophy	1 (12.5)
Incomplete	
>50% of the adjacent cartilage	1 (12.5)
<50% of the adjacent cartilage	1 (12.5)
Subchondral bone exposed	0 (0)
2. Integration to border zone	
Complete	6 (75.0)
Incomplete	
Demarcating border visible (split-like)	1 (12.5)
Defect visible	
<50% of the length of the repair tissue	1 (12.5)
>50% of the length of the repair tissue	0 (0)
3. Surface of the repair tissue	
Surface intact	6 (75.0)
Surface damaged	
<50% of repair tissue depth	1 (12.5)
>50% of repair tissue depth or total degeneration	1 (12.5)
4. Structure of the repair tissue	
Homogenous	4 (50.0)
Inhomogenous or cleft formation	4 (50.0)
5. Signal intensity of the repair tissue	
Dual T2-FSE	
Isointense	6 (75.0)
Moderately hyperintense	1 (12.5)
Markedly hypointense	1 (12.5)
6. Subchondral Lamina	
Intact	6 (75.0)
Non-intact	2 (25.0)
7. Subchondral bone	
Intact	6 (75.0)
Non-intact	2 (25.0)
8. Adhesions	
No	8 (100.0)
Yes	0 (0)
9. Effusion	
No	8 (100.0)
Yes	0 (0)

The structural outcomes for the regenerated cartilage were evaluated rigorously. Preoperative arthroscopic images, laser-induced photoacoustic (LIPA) evaluation, and chondrocyte sheet transplantation of a representative patient are shown in Movie 1 in the Supplementary information. Postoperative arthroscopic images, LIPA evaluation, and biopsy of the regenerated cartilage in the same patient are shown in Movie 2 of the Supplementary information. For the LIPA evaluation, the viscoelastic characteristics of regenerated cartilage are represented as the ratios relative to those of normal cartilage in the same joint; these values were calculated to be 0.87–1.06 (Table 1).

Histological evaluations of all patient biopsies revealed strong staining for Safranin O and expression of type II collagen (COL2); samples from three patients showed type I collagen (COL1) expression in the superficial regions. Although the degree to which regeneration occurred varied between patients, these results suggest regeneration of hyaline cartilage (Fig. 3a and Table 1).

### Predicting clinical and structural outcomes by analyzing cell sheets

**Fig. 4:**
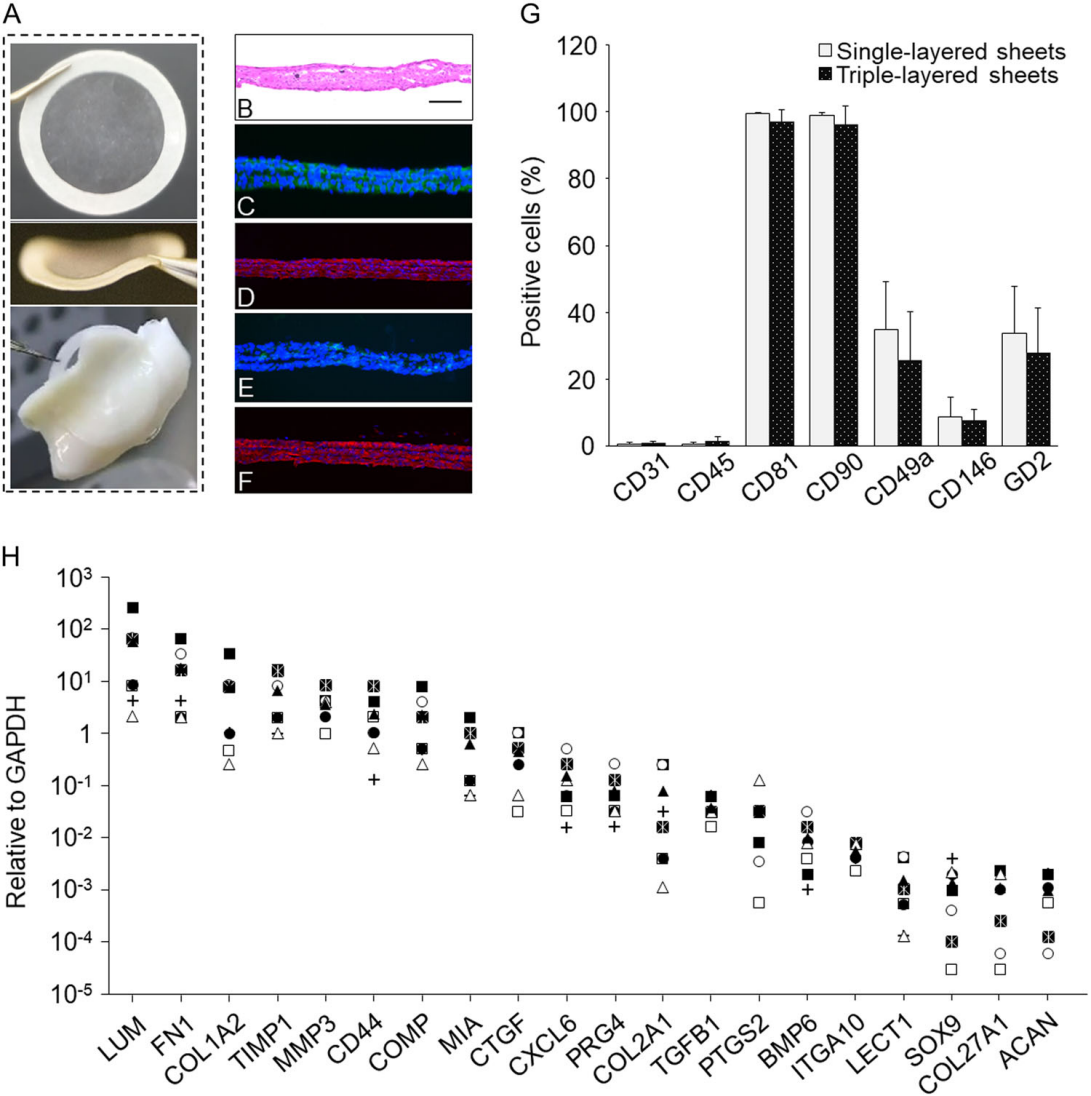
Properties of layered chondrocyte sheets are indicated. **a** Layered chondrocyte sheets were handled using a circular PVDF support membrane. The thin sheets attached readily to the surfaces of cartilage tissue. **b**–**f** Representative images show histological staining with hematoxylin and eosin. **b** Representative image showing histological staining with hematoxylin and eosin; scale bar = 100 μm. Immunohistological staining of layered chondrocyte sheets. **c** fibronectin, **d** type I collagen, **e** type II collagen, and **f** aggrecan. **g** Flow cytometric analysis of surface markers for chondrocytes contained in the single- and triple-layered sheets. The two kinds of cell sheets, single- and triple-layered sheets, which had been prepared for transplantation, were sacrificed and dispersed enzymatically before layering after 14 days of culture and on day 1 before transplantation, respectively. The cells sheets were analyzed by single-color staining. Data are expressed as the mean ± SD of the percentage of surface marker-expressing cells from eight independent sheets manufactured from each patient. Chondrocytes were positive for CD81 and CD90 and negative for CD31 and CD45. Staining for CD49a, CD146, and GD2 differed between patients. **h** The gene expression profile of transplanted sheets from each patient was analyzed using qPCR for cartilage-related genes, and the results are reported relative to the expression of *GAPDH*

The layered chondrocyte sheets can be handled using a circular white support membrane of polyvinylidene difluoride (PVDF) and appear as thin membranes that exhibit strong adhesive properties (Fig. 4a). The multilayered structure was confirmed by histological staining (Fig. 4b). Immunostaining showed that the sheets strongly expressed fibronectin (FN), COL1, and aggrecan (ACAN), and weakly expressed COL2 (Fig. 4c–f). The chondrocytes in the sheets were dedifferentiated and the properties of chondrocyte sheets were different from those of hyaline cartilage.

Almost all chondrocytes from these sheets were positive for CD81 and CD90. CD49a- and GD2-positive cells were present at a frequency of 20–30% and CD146-positive cells at a frequency of 10%. There were almost no CD31- and CD45-positive cells (Fig. 4g).

Quantitative polymerase chain reaction (qPCR) analysis of the genes expressed by the layered chondrocyte sheets revealed widespread expression of cartilage-related genes of interest, However, the expression levels of ACAN, SOX9, and COL2A1 decreased relative to GAPDH (Fig. 4h).

Predictive gene marker sets for postoperative clinical and structural outcomes were identified based on correlation coefficient analysis. The marker sets were as follows: for overall KOOS (Fig. 3d) *ACAN*, *CSGALNACT1*, *BMP6*, *PTGS2*, *POU5F1*, and *CXCL6*; for the LKS (Fig. 3e) *GATA6*, *LECT1*, *GDF5*, *ADAMTS5*, *SP7*, and *MATN2*; and for the Osteoarthritis Research Society International (OARSI) histological score (Fig. 3f) *ACAN*, *BMP6*, *POU5F1*, *CXCL6*, *VEGFA*, and *PTGS2*. Cronbach’s alpha coefficients of these data sets were 0.73 for KOOS, 0.93 for LKS, and 0.65 for OARSI.

## Discussion

OAK-related cartilage defects develop over many years and often require multiple therapies to treat coexisting pathological conditions such as malalignment and ligamentous and meniscal disorders. Therefore, complying with regulations that require clear evidence of the add-on effect of each treatment makes the development of new therapies particularly challenging. Here, we challenged the nature of these regulations by applying autologous chondrocyte transplantation combined with conventional surgical treatments and rigorous follow-up procedures in the first human clinical study of regenerative medicine applied to OAK in Japan. The properties of transplanted chondrocyte sheets were also evaluated thoroughly using gene expression analysis to investigate the possibility of predicting clinical and structural outcomes.

Our study has four limitations. First, the study included only eight patients, who presented with various preoperative conditions. Three patients with post-traumatic OA underwent anterior cruciate ligament (ACL) reconstruction, and five patients with general OA underwent open-wedge high tibial osteotomy (OWHTO). These associated surgeries may have affected the outcomes of the therapy. Second, we observed promising outcomes for the use of layered chondrocyte sheets over at least 3 years, but longer-term observations are needed to evaluate this new combination therapy fully. Third, we had to adopt a two-stage surgical approach, first to harvest healthy cartilage and synovium, and second to transplant the layered chondrocyte sheets. Although regenerative medicine is commonly performed using autologous cells, this requires the sacrifice of healthy cartilage at a nonloading site of the knee, which comprises a limited area. Therefore, it is difficult to perform this procedure more than twice. Finally, our clinical study was designed as a single-arm, nonrandomized, and uncontrolled study.

For this small initial longitudinal series, the combination therapy was effective as assessed by MRI, arthroscopy, histology, and the clinical outcomes assessed by the KOOS and LKS. The marker gene sets identified from autologous chondrocyte sheets may be effective predictors of the outcomes as measured by the overall KOOS, LKS, and OARSI histological scores, and may be potential alternative markers for evaluating OAK treatment. Determining the true effectiveness of this therapy for OAK will require long-term follow-up and strict comparative studies. A previous prospective clinical study by Wakitani et al.^23^ on bone marrow mesenchymal cell transplantation with a COL1 gel and periosteum patch combined with high tibial osteotomy for OAK. Although the clinical improvement did not differ significantly between the cell-transplanted and cell-free groups in that study, the arthroscopic and histological grading scores were better in the cell-transplanted group. These results show that it is very difficult to show an add-on effect of cell transplantation in OAK patients.

In this clinical study, we demonstrated evidence of hyaline cartilage repair after chondrocyte sheet transplantation in humans. A previous study by Sekiya et al.^24^ reporting the clinical outcomes of ten patients with knee cartilage defects treated using transplantation of synovial stem cells showed that most histological sections contained hyaline cartilage in the deep zone and fibrous tissue in the middle and surface zones. By contrast, we have previously reported that pain-alleviating effects and hyaline cartilage repair were achieved after transplantation of layered chondrocyte sheets but not after transplantation with synovial cells.^12^ These findings suggest that the humoral factors produced by layered chondrocyte sheets may contribute to cartilaginous tissue repair and regeneration.^16^ In the cases of these animal experiments, we always create the cartilage (osteochondral) defects to be 8 mm in diameter and at 5 mm in depth in minipigs, 5 mm in diameter and at 3 mm in depth in rabbits, and 2 mm in diameter and at 1 mm in depth in rats. We confirmed bleeding from the subchondral bone because of the thin cartilage in these animals. These conditions are considered to be marrow stimulating, as in a clinical procedure. Therefore, we have not needed to create additional holes at the sites of the defects.

This study was also designed as a feasibility study to overcome the conventional requirements of OAK treatment, to show the total effectiveness of the combined treatment, and to identify alternative markers of clinical endpoints, but not to identify the add-on effect of each treatment component. Because OAK is a heterogeneous disease characterized by variable clinical features, biochemical and genetic characteristics, and responses to treatments, control groups for comparative studies always include individuals with heterogeneous conditions. In this small initial longitudinal case series, we demonstrated that the expression of selected marker gene sets in autologous transplanted chondrocyte sheets derived from each patient may be effective predictors of the clinical and structural outcomes of this new therapy for OAK. To our knowledge, this is the first study to identify gene sets that may predict the clinical and structural outcomes of regenerative medicine. The chondrocytes contained in the sheets have dedifferentiated (Fig. 4c–f, h), but our previous data also showed that humeral factors secreted from the sheets may contribute to cartilage repair and regeneration.^16,25^

From our study results, we propose that our procedure using chondrocyte sheets has three potential advantages. First, for safety, we use only autologous cells without any foreign materials. Second, chondrocyte sheets generated using temperature-responsive culture inserts express FN strongly and have excellent adhesive properties, meaning that the periosteum is not required. Third, several types of animal experiments have demonstrated that hyaline cartilage repair may be possible using chondrocyte sheets,^12–15^ and the biopsy samples of all patients in the present study demonstrate that this may also be possible in humans.

We have often experienced difficulties in the precise evaluation of the efficacy of OAK treatment because OAK is a heterogeneous and slowly progressive disease. For this small initial longitudinal series, the combination therapy for OAK was effective, as assessed by MRI, arthroscopy, viscoelasticity, histology, and clinical outcomes such as the KOOS and LKS.

Medial osteophyte progression was observed only in this patient (Fig. 2c–e), and we have not identified any effects or clinical symptoms caused by this osteophyte. However, Felson et al.^26^ reported that osteophytes are strongly associated with malalignment to the side of the osteophyte and that malalignment is a potential risk factor for progression of OA. We consider it unlikely that this patient’s osteophyte progression reflects malalignment after the OWHTO because the patient is quite active in daily life. We plan careful long-term follow-up of this patient.

In addition, the expression of marker gene sets in autologous chondrocyte sheets appeared to be predictive of the outcomes of the LKS and was weakly predictive of the overall KOOS and OARSI histological scores. A longitudinal prospective study of therapy using autologous cells may be able to determine whether the gene sets identified in the regenerative product can predict the longer-term clinical and structural outcomes of this treatment. The gene sets that would be predict the structural outcomes of histological scores may provide alternative markers for evaluating OAK treatment and predicting the long-term prognosis of the therapy.

Although the method used in our clinical study is challenging for the regulatory authorities, we believe it is essential to continue investigating the outcomes in more patients with OAK. Determining the true effectiveness of this therapy for OAK requires longer-term follow-up and strict comparative studies.

## Methods

Each author certifies that his institution approved the human protocol for this clinical study that all investigations were conducted in conformity with ethical principles of research, and that informed consent for participation in the study was obtained. Investigations were performed at Tokai University School of Medicine, Isehara, Japan.

### Study design and setting

Our clinical study was designed as a single-arm, nonrandomized, uncontrolled study. This study (UMIN Clinical Trials Registry, UMIN000006650) was approved by the Institutional Review Board of the Tokai University School of Medicine and by the Ministry of Health, Labor, and Welfare of Japan. The written informed consent was obtained from all participants.

### Participants/study subjects

Eligible patients were aged 20 to 60 years and had a symptomatic cartilage lesion involved in OAK. The inclusion criteria included cartilage defects (of the condyle of the femur and/or the patellofemoral joint) that would traditionally have been indications for marrow stimulation techniques or osteochondral autografts that were equivalent to Outerbridge grade III or IV and sized less than 4.2 cm^2^/defect. The exclusion criteria included difficulty in obtaining informed consent from the patient; complications hindering surgery under general anesthesia or affecting knee surgery; problematic infectious disease (including being positive for hepatitis B virus, hepatitis C virus, human immunodeficiency virus, human T-cell leukemia virus-1, or the fluorescent treponemal antibody-absorption test); and a history of rheumatoid arthritis or other systemic inflammatory disease. The final decision about entry into the clinical study was made during arthroscopic evaluation 3–4 weeks before the operation.

Ten patients who had cartilage defects categorized arthroscopically as Outerbridge grade III or IV were enrolled, and eight patients received the therapy. Two patients were excluded because we were unable to collect a sufficient volume of cartilage in one and were unable to generate layered chondrocyte sheets from the second.

The first patient was enrolled in November 2011 and the last in August 2013. All patients completed follow-up of at least 3 years. The median age of the patients was 48 years (range, 30–59 years), the median duration of symptoms was 11.8 years (range, 5–20 years), the median size of each cartilage defect was 3.44 cm^2^ (range, 2.25–4.10 cm^2^/defect), and the median follow-up was 54 months (range, 36–67 months). For patients meeting the inclusion criteria, a LIPA method was used to assess cartilage viscoelasticity,^27–29^ and cartilage and synovial tissues were collected for the fabrication of chondrocyte sheets. Details of the eight patients who received the combination therapy are shown in Table 1.

### Surgery and rehabilitation

We designed a combination therapy in which conventional surgical treatment for OAK, covered by National Health Insurance, was followed by the RMSC method (Fig. 1b). Eight patients with OAK accompanied by cartilage defects of up to 4.2 cm^2^ received the therapy. This size of the defect was recommended by the Ministry of Health, Labor and Welfare as being within the size of a cell sheet. Three patients with post-traumatic OA underwent arthroscopic ACL reconstruction,^30^ and five patients with general OA underwent OWHTO.^31^ After these conventional surgeries, the knee joint was opened using the parapatellar medial approach. The medial femoral condyle, medial tibial plateau, and patellofemoral joint were observed. In all patients, the articular cartilage on these lesions had been lost, and the subchondral bone was often eburnated (Supplementary Movie 1). All cartilage defects were defined as Outerbridge grade III or IV.^32^ A rigorous evaluation protocol was designed to determine the safety and efficacy of the therapy (Fig. 1c). Pre- and postoperative images of a representative patient who underwent the therapy are shown in Fig. 2.

All patients were immobilized immediately after surgery using a plaster splint that was maintained for 2 weeks at 20-degree flexion of the knee so as not to disturb the transplanted sheets. Patients then started range-of-motion exercise, partial weight bearing at 2 weeks, and full weight bearing at 4 weeks after surgery. In general, low-impact activities started at 6 months, and high-impact activities were allowed at 8 months.

### Variables, outcome measures, data sources, and bias

Imaging assessment was performed using X-ray photographs and MRI. X-ray photographs were examined for the knee alignment, the condition of the subchondral bone, and the progression of OAK. Progress was evaluated using the Kellgren–Lawrence grading scale pre- and postoperatively. MRI examinations were performed using an Achieva 3.0-T TX scanner (Philips Healthcare, Best, The Netherlands), within a TX SENSE Knee eight-channel coil (Philips Healthcare). The images were taken with a 10-degree flexion of the knee. Sagittal and coronal MR images were used to evaluate the cartilage defect areas pre- and postoperatively.

For evaluation of the therapy, we used the MOCART method described by Marlovits et al.^33^ Table 2 shows the variables included in the MOCART. Arthroscopic evaluation of cartilage defects for condition, size, and Outerbridge grade was performed pre- and postoperatively. The LIPA method was used to evaluate the viscoelastic properties of cartilage pre- and postoperatively (Supplementary Movies 1 and 2). The use of LIPA was approved by the Institutional Review Board for Clinical Research at Tokai University, and the method has been applied clinically to evaluate the mechanical properties of cartilage in patients.^29^

Clinical outcomes were evaluated with the patient-oriented KOOS^34^ and the LKS^35^ preoperatively and 1, 3, 6, 12, 24, and 36 months postoperatively.

Histological outcomes were evaluated 12-months postoperatively using an arthroscopically performed biopsy taken from near the center of the regenerated cartilage. Samples were embedded in paraffin wax, and 3 μm histological sections were cut, deparaffinized, stained with Safranin O, and immunostained for COL1 and COL2 using previously reported methods.^13^ The OARSI histological score was assessed independently by three trained orthopedic surgeons. Microscopic scoring of the cartilage was evaluated using the method described by Little et al.^36^ who provided representative images for each score. There were minimal variations in scoring between the three scorers. The Mankin score^37^ was obtained similarly. Structural compromise (0–6 points), loss of matrix staining (0–4 points), cellularity anomalies (0–3 points), and violation of tidemark integrity (0 or 1 point) were graded using this scale, with normal cartilage given a score of 0 out of 14 points.

### Fabrication of triple-layered chondrocyte sheets

Triple-layered chondrocyte sheets were fabricated using previously described methods.^16,17,20^ Layered chondrocyte sheets were manufactured for the eight patients at the cell-processing center in our hospital, and their physical and functional characteristics were analyzed to identify predictors of clinical effectiveness. In brief, the collected chondrocytes and synovial cells from each patient were passed through a cell strainer (BD Biosciences, Franklin Lakes, NJ, USA) with a pore size of 100 μm, and the cells were retrieved by centrifugation. The synovial cells were seeded into six-well plates (BD Biosciences) at 1 × 10^4^ cells/cm^2^, and the chondrocytes were seeded into temperature-responsive culture inserts (4.2 cm^2^; CellSeed Inc., Tokyo, Japan) at 5 × 10^4^ cells/cm^2^ (Fig. 1a). The chondrocytes and synovial cells were cocultured for 14–17 days in Dulbecco’s minimal essential medium/F12 supplemented with 20% fetal bovine serum and 1% antibiotic–antimycotic solution at 37 °C in 5% CO_2_ and 95% air. From day 4, 50 μg/ml ascorbic acid (Wako Pure Chemical Industries, Ltd., Osaka, Japan) was added to the culture medium. After 14–17 days of coculture, the chondrocyte sheets on temperature-responsive culture inserts were removed from the incubator and left at 20 °C for 30 min (Fig. 1a). After removal of the culture medium, a PVDF membrane was used to retrieve each chondrocyte sheet (Fig. 4a) as previously described.^38^ Briefly, a chondrocyte sheet was covered with a PVDF membrane, and the edges of the sheet were folded onto the membrane. The sheet and membrane were harvested carefully as one unit and placed on top of another chondrocyte sheet. The second and third sheets were harvested similarly and used to fabricate triple-layered chondrocyte sheets. A cell strainer (BD Biosciences) was placed on top of the layered sheet to prevent floating. The triple-layered chondrocyte sheets were cultured for a further 7–8 days in culture dishes.

### Flow cytometric characterization of chondrocyte sheets

Flow cytometric analysis of chondrocyte sheets was performed on single-layer sheets before layering and on triple-layered sheets on the day before transplantation. Chondrocyte sheets were digested with TrypLE Express (Thermo Fisher Scientific, Waltham, MA, USA) at 37 °C for 15 min and then incubated with 0.25 mg/ml collagenase-P (Roche, Basel, Switzerland) at 37 °C for 30 min. The dispersed cells were washed with Ca^++^/Mg^++^-free phosphate-buffered saline (PBS) containing 0.2% human serum albumin (Sigma-Aldrich, St. Louis, MO, USA) and 1 mM ethylenediaminetetraacetic acid (EDTA; Wako Pure Chemical Industries, Ltd.), and then immunostained with the following antibodies: CD31–fluorescein isothiocyanate (FITC) (clone: 5.6E) and CD45–FITC (clone: J.33) from Beckman Coulter, Inc. (La Brea, CA, USA); CD81–allophycocyanin (APC) (clone: JS-81), CD90–APC (clone: 5E10), CD49a–phycoerythrin (PE) (clone: SR84), and disialoganglioside GD2 (clone: 14.G2a) from BD Biosciences; and CD146–PE (clone: F4-35H7 (S-Endo 1)) from BioCytex (Marseille, France). Fluorochrome-labeled mouse IgG1 antibody (clone: 679.1Mc7, Beckman Coulter) was used as a negative control and FITC-conjugated goat anti-mouse IgG (BD Biosciences) was used as the secondary antibody. Stained cells were analyzed using a FACSVantage flow cytometer (BD Biosciences).

### Immunohistochemical characterization of chondrocyte sheets

Cryosections (20-μm thick) of chondrocyte sheets were fixed with 4% paraformaldehyde in phosphate buffer (PB) and washed three times with PBS. For histological analysis, sections were stained with hematoxylin and eosin. For immunohistochemistry, sections were blocked with 5% normal goat serum and 0.3% Triton X-100 in PB for 30 min. The sections were then incubated with primary antibodies to human COL1 (SouthernBiotech, Birmingham, AL, USA; dilution 1:200), COL2 (Kyowa Pharma Chemical Co., Ltd., Toyama, Japan; dilution 1:200), FN (Merck Millipore, Darmstadt, Germany; dilution 1:500), or ACAN (R&D Systems, Minneapolis, MN, USA; dilution 1:10) at 4 °C overnight. The sections were then washed and incubated at room temperature for 1 h with the secondary antibody Alexa Fluor 488-conjugated goat anti-mouse Ig (Thermo Fisher Scientific) for COL2 and FN, or donkey Alexa Fluor 546-conjugated anti-goat Ig (Thermo Fisher Scientific) for COL1 and ACAN. After immunostaining, the slides were stained with 4′,6-diamidino-2-phenylindole (Vector Laboratories, Burlingame, CA, USA) and observed under a BZ-9000 fluorescence microscope (Keyence Corp., Osaka, Japan).

### Gene expression analysis of chondrocyte sheets

Chondrocyte sheets from the day before transplantation were disrupted in TRIzol Reagent (Life Technologies, Carlsbad, CA, USA), and total RNA was isolated using an SV Total RNA Isolation System (Promega Corp., Fitchburg, WI, USA). Total RNA was converted to cDNA using oligo(dT)16 primers and MuLV Reverse Transcriptase (both from Applied Biosystems, Waltham, MA, USA) at 48 °C for 30 min. A TaqMan PreAmp Master Mix Kit (Applied Biosystems) was used to preamplify the cDNA. TaqMan gene expression assay kits (Applied Biosystems) including fluorescent probes and forward–reverse primers were diluted with Tris-EDTA buffer (1×) according to the manufacturer’s instructions to obtain a final concentration of 0.2× for the pooled assay mix. TaqMan qPCR was performed using the 7500 qPCR System (Thermo Fisher Scientific). The relative expression values for each gene (–ΔCt values) were calculated using *GAPDH* as the internal control. qPCR was not performed for one of the eight patients because only a very small amount of total RNA could be isolated after the extraction process.

Predictive gene marker sets were selected from the 45 genes and control genes examined (*GAPDH* and *ACTB*) (Table 3 in Supplementary information) as those whose expression correlated with the OARSI histological score or the overall KOOS and LKS 12 months postoperatively. First, 41 of 45 genes with normalized ΔCt values greater than –15 cycles were identified as having detectable gene expression without more than 30% missing values. Then, for each outcome assessment, Pearson correlation coefficients were calculated between the expression level of each of these 41 genes and the individual scores (KOOS, LKS, and OARSI histological score) using the leave-one-out process, and the genes were ranked from highest to lowest for the absolute values of the coefficients. From this, a matrix of seven sets of Pearson correlation coefficients was calculated. Five representative genes were selected independently as predictive markers for each of the outcome assessments by selecting the top five genes in rank order, and listed by their probability of appearance. Finally, six gene sets with the highest probability of appearance were selected as the predictive markers. The gene expression score (GES) was calculated by weighted voting of the appearance probability of each predictive marker gene based on linear functions (GES = ∑ (*p* × ΔCt)).

### Statistical analysis

Clinical outcomes were assessed using the KOOS and LKS according to the protocol (Fig. 1c). One-way analysis of variance with a post hoc Holm–Bonferroni test was used to identify differences between pre- and postoperative scores for clinical outcomes (*n* = 8). *P*-values < 0.05 were considered to be significant.

### Reporting summary

Further information on experimental design is available in the Nature Research Reporting Summary linked to this article.

## Supplementary information

Supplementary file

Supplementary Data 1

Supplementary Data 2

Supplementary Movie 1.(AVI)

Supplementary Movie 2.(AVI)

Reporting Summary

## Data Availability

The data sets generated and/or analyzed during the current study are available from the corresponding author on reasonable request.

## Supplementary information

**Supplementary information** accompanies the paper on the *npj Regenerative Medicine* website (10.1038/s41536-019-0069-4).
