# Supplementary material for: Combined surgery and chondrocyte cell-sheet transplantation improves clinical and structural outcomes in knee osteoarthritis
Source: NPJ Regen Med. 2019 Feb 21;4:4. doi: 10.1038/s41536-019-0069-4 (PMC6384900; doi:10.1038/s41536-019-0069-4)
Supplement: Supplementary file 1 — Supplementary file [file 41536_2019_69_MOESM1_ESM.pdf]

- Supplementary Table 3. Probes used in qPCR

- Supplementary Data 3. Figures and raw data for Fig 4.

- Clinical study for knee joint treatment using cell sheets: Study protocol

Supplementary Table 3. Probes used in qPCR

Gene Symbol	Assay ID	Reference Sequence
ACAN	Hs00153936_m1	NM_013227.3;NM_001135.3
ACTB	Hs01060665_g1	NM_001101.3
ADAMTS5	Hs00199841_m1	NM_007038.3
BGLAP	Hs01587814_g1	NM_199173.4
BMP6	Hs01099599_gH	NM_001718.4
CD44	Hs01075861_m1	NM_000610.3;NM_001202555.1;NM_001202556.1 NM_001001392.1;NM_001001391.1;NM_001001390.1 NM_001001389.1
COL1A1	Hs01076775_g1	NM_000088.3
COL1A2	Hs01028971_m1	NM_000089.3
COL2A1	Hs01060356_g1	NM_033150.2;NM_001844.4
COL6A1	Hs01095599_g1	NM_001848.2
COL9A1	Hs00932129_m1	NM_078485.3;NM_001851.4
COL10A1	Hs00166657_m1	NM_000493.3
COL11A2	Hs00899185_g1	NM_080681.2;NM_080679.2;NM_080680.2
COL27A1	Hs00259829_m1	NM_032888.2
COMP	Hs01572837_g1	NM_000095.2
CSGALNACT1	Hs00218054_m1	NM_018371.4;NR_024040.1;NM_001130518.1
CTGF	Hs01026927_g1	NM_001901.2
CXCL6	Hs00605742_g1	NM_002993.3
CXCR4	Hs00607978_s1	NM_003467.2;NM_001008540.1
ECM2	Hs00154821_m1	NM_001197296.1;NM_001393.3;NM_001197295.1
FN1	Hs01549970_g1	NM_212482.1;NM_002026.2;NM_212476.1 NM_212478.1;NM_212474.1
GAPDH	Hs02758991_g1	NM_002046.4;NM_001256799.1
GATA6	Hs00232018_m1	NM_005257.4
GDF5	Hs00167060_m1	NM_000557.2
ITGA10	Hs01006923_g1	NM_003637.3
LECT1	Hs00993254_m1	NM_001011705.1;NM_007015.2
LIN28A	Hs00702808_s1	NM_024674.4
LUM	Hs00929860_m1	NM_002345.3
MATN2	Hs01051833_g1	NM_030583.2;NM_002380.3
MCAM	Hs00174838_m1	NM_006500.2
MGP	Hs00969490_m1	NM_000900.3;NM_001190839.1

MIA	Hs00197954_m1	NM_006533.3;NM_001202553.1;NR_037775.1
MMP3	Hs00968305_m1	NM_002422.3
MMP13	Hs00233992_m1	NM_002427.3
POU5F1	Hs03005111_g1	NM_002701.5
PRG4	Hs00981633_m1	NM_005807.3;NM_001127710.1;NM_001127708.1<br>NM_001127709.1
PTGS2	Hs00153133_m1	NM_000963.2
RUNX2	Hs00231692_m1	NM_001015051.3;NM_001278478.1;NM_001024630.3
SERPINF1	Hs01106934_m1	NM_002615.5
SNX19	Hs01040307_g1	NM_014758.2
SOX5	Hs00753050_s1	NM_001261414.1;NM_006940.4;NM_001261415.1<br>NM_178010.2;NM_152989.3
SOX6	Hs00264525_m1	NM_033326.3;NM_017508.2;NM_001145819.1<br>NM_001145811.1
SOX9	Hs01001343_g1	NM_000346.3
SP7	Hs01866874_s1	NM_152860.1;NM_001173467.1
TGFB1	Hs00998133_m1	NM_000660.4
TIMP1	Hs00171558_m1	NM_003254.2
VEGFA	Hs00900055_m1	NM_001025366.2;NM_001025367.2;NM_001025368.2<br>NM_001025369.2;NM_001025370.2;NM_001033756.2<br>NM_001171622.1;NM_001171623.1;NM_001171624.1<br>NM_001171625.1;NM_001171626.1;NM_001171627.1<br>NM_001171628.1;NM_001171629.1;NM_001171630.1<br>NM_001204384.1;NM_001204385.1;NM_001287044.1<br>NM_001317010.1;NM_003376.5

Supplementary Data 3. Figures and raw data for Fig 4.

Case 1

Single-layered sheets (1)

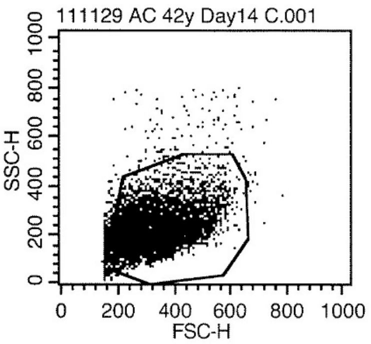

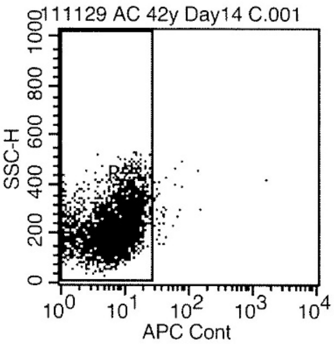

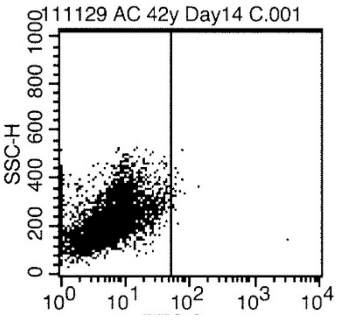

Quad	Events	% Gated
UL	0	0.00
UR	0	0.00
LL	4837	99.53
LR	23	0.47

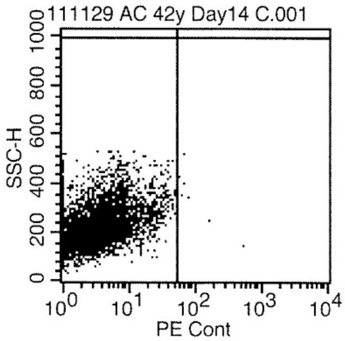

Quad	Events	% Gated
UL	0	0.00
UR	0	0.00
LL	4851	99.81
LR	9	0.19

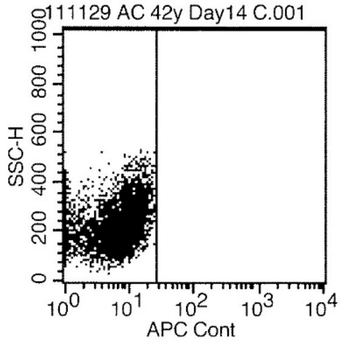

Quad	Events	% Gated
UL	0	0.00
UR	0	0.00
LL	4856	99.92
LR	4	0.08

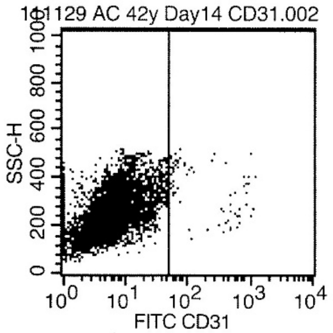

Quad	Events	% Gated
UL	0	0.00
UR	0	0.00
LL	5517	98.75
LR	70	1.25

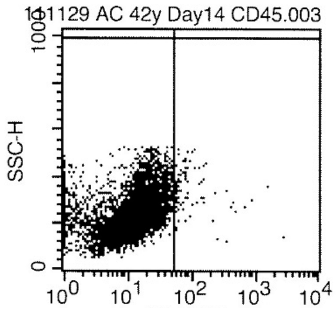

Quad	Events	% Gated
UL	0	0.00
UR	0	0.00
LL	5348	98.07
LR	105	1.93

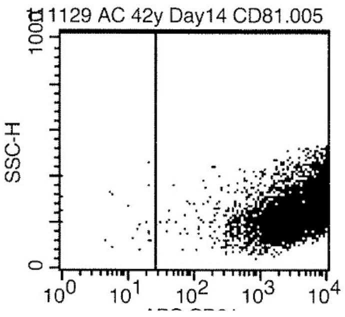

Quad	Events	% Gated
UL	0	0.00
UR	0	0.00
LL	20	0.39
LR	5160	99.61

Case 1
Single-layered sheets (2)

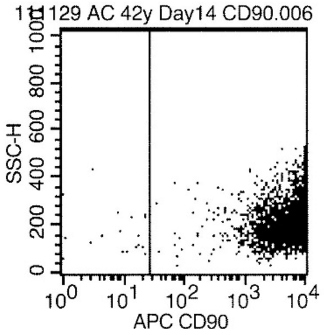

Quad	Events	% Gated
UL	0	0.00
UR	0	0.00
LL	18	0.40
LR	4432	99.60

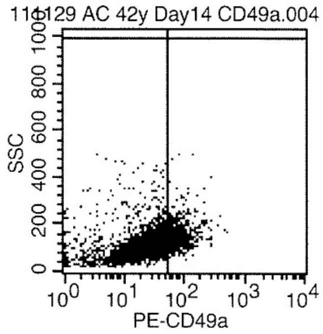

Quad	Events	% Gated
UL	0	0.00
UR	0	0.00
LL	3469	83.09
LR	706	16.91

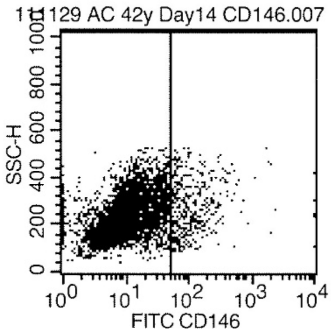

Quad	Events	% Gated
UL	0	0.00
UR	0	0.00
LL	4519	91.57
LR	416	8.43

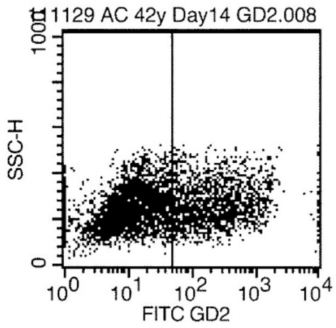

Quad	Events	% Gated
UL	0	0.00
UR	0	0.00
LL	2966	66.21
LR	1514	33.79

Case 1
Triple-layered sheets (1)

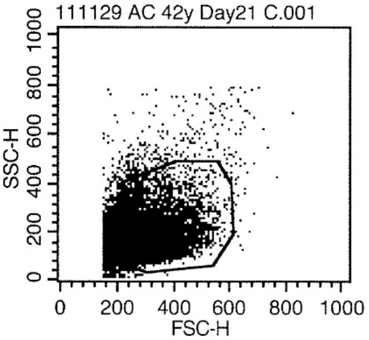

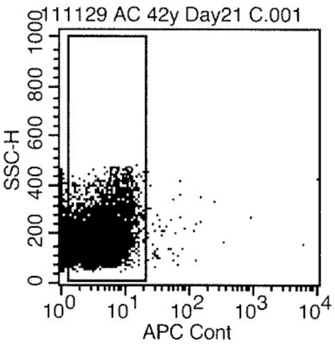

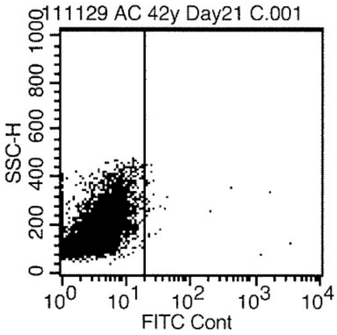

Quad	Events	% Gated
UL	0	0.00
UR	0	0.00
LL	5688	99.32
LR	39	0.68

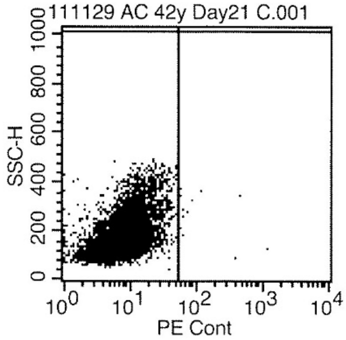

Quad	Events	% Gated
UL	0	0.00
UR	0	0.00
LL	5714	99.77
LR	13	0.23

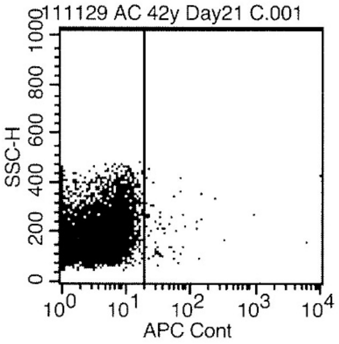

Quad	Events	% Gated
UL	0	0.00
UR	0	0.00
LL	7862	99.14
LR	68	0.86

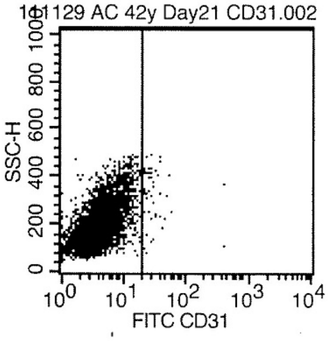

Quad	Events	% Gated
UL	0	0.00
UR	0	0.00
LL	3661	98.47
LR	57	1.53

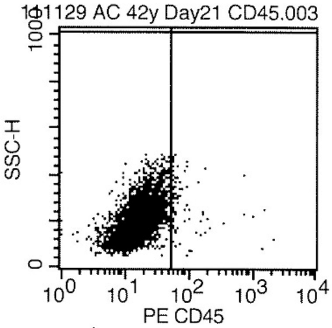

Quad	Events	% Gated
UL	0	0.00
UR	0	0.00
LL	3362	97.36
LR	91	2.64

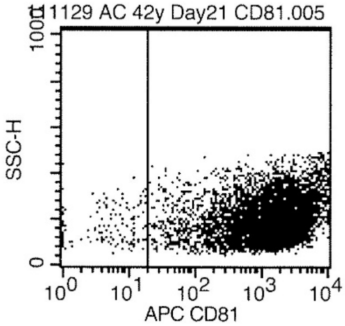

Quad	Events	% Gated
UL	0	0.00
UR	0	0.00
LL	139	2.43
LR	5581	97.57

Case 1
Triple-layered sheets (2)

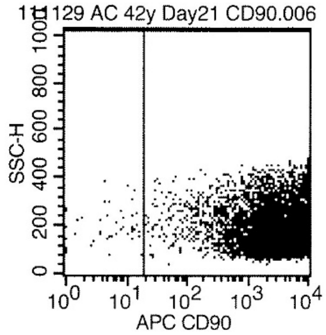

Quad	Events	% Gated
UL	0	0.00
UR	0	0.00
LL	46	0.79
LR	5779	99.21

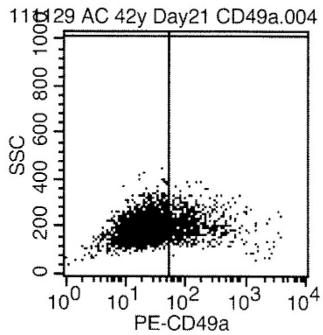

Quad	Events	% Gated
UL	0	0.00
UR	0	0.00
LL	2424	79.32
LR	632	20.68

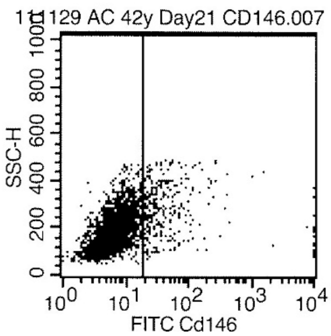

Quad	Events	% Gated
UL	0	0.00
UR	0	0.00
LL	2283	87.57
LR	324	12.43

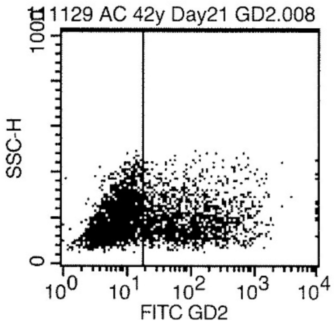

Quad	Events	% Gated
UL	0	0.00
UR	0	0.00
LL	2493	63.89
LR	1409	36.11

Case 2
Single-layered sheets (1)

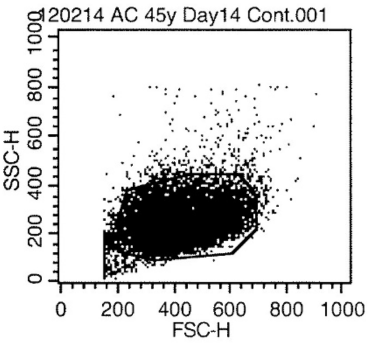

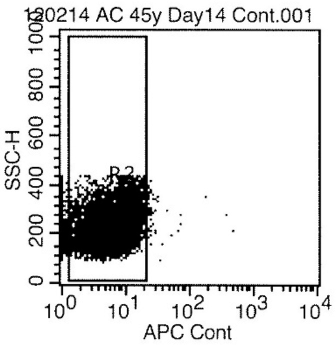

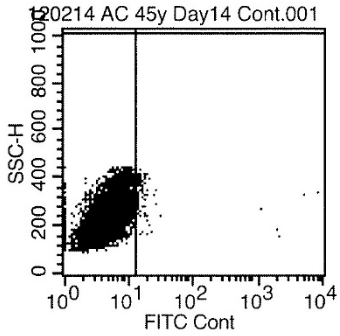

Quad	Events	% Gated
UL	0	0.00
UR	0	0.00
LL	9252	99.03
LR	91	0.97

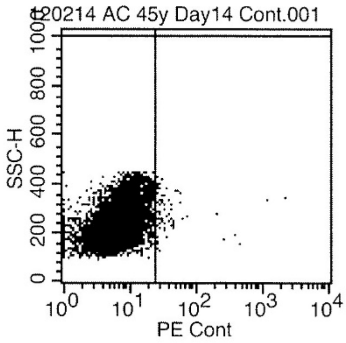

Quad	Events	% Gated
UL	0	0.00
UR	0	0.00
LL	9266	99.18
LR	77	0.82

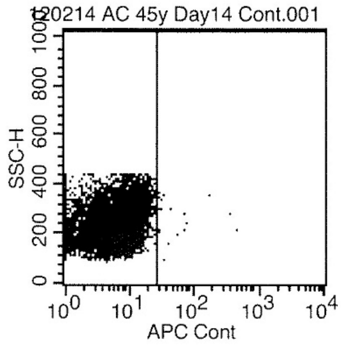

Quad	Events	% Gated
UL	0	0.00
UR	0	0.00
LL	10599	99.82
LR	19	0.18

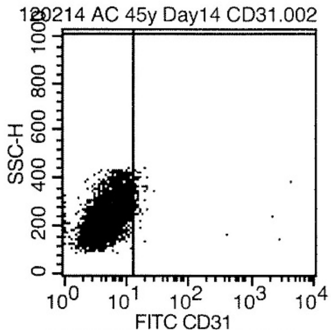

Quad	Events	% Gated
UL	0	0.00
UR	0	0.00
LL	6835	99.04
LR	66	0.96

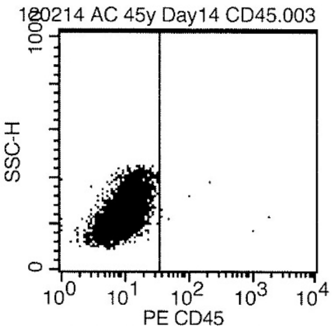

Quad	Events	% Gated
UL	0	0.00
UR	0	0.00
LL	7059	99.83
LR	12	0.17

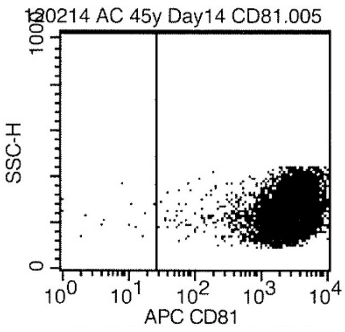

Quad	Events	% Gated
UL	0	0.00
UR	0	0.00
LL	13	0.12
LR	10404	99.88

Case 2
Single-layered sheets (2)

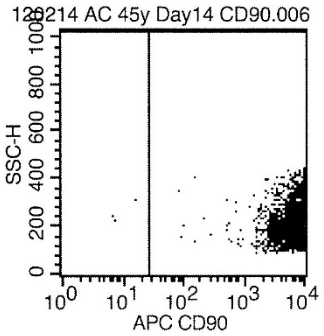

Quad	Events	% Gated
UL	0	0.00
UR	0	0.00
LL	3	0.03
LR	9747	99.97

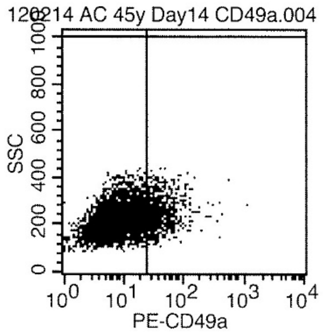

Quad	Events	% Gated
UL	0	0.00
UR	0	0.00
LL	3015	81.93
LR	665	18.07

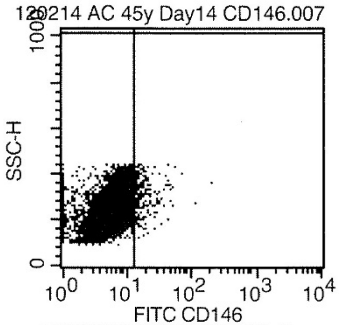

Quad	Events	% Gated
UL	0	0.00
UR	0	0.00
LL	6473	92.23
LR	545	7.77

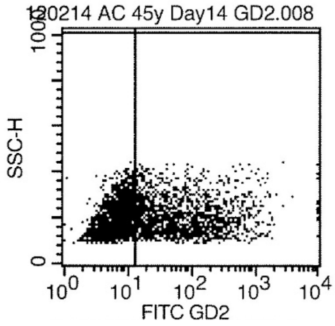

Quad	Events	% Gated
UL	0	0.00
UR	0	0.00
LL	1942	55.80
LR	1538	44.20

Case 2
Triple-layered sheets (1)

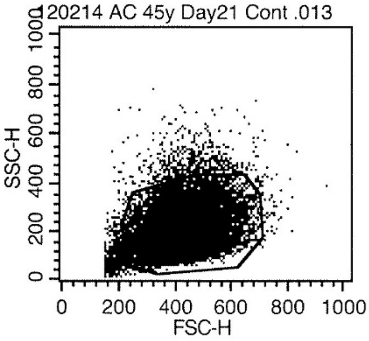

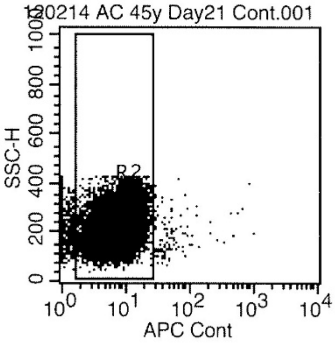

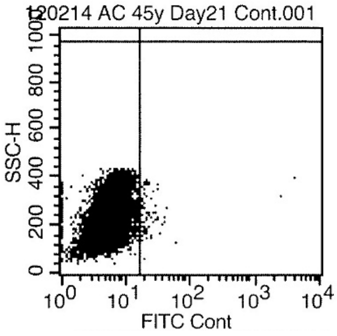

Quad	Events	% Gated
UL	0	0.00
UR	0	0.00
LL	9831	99.14
LR	85	0.86

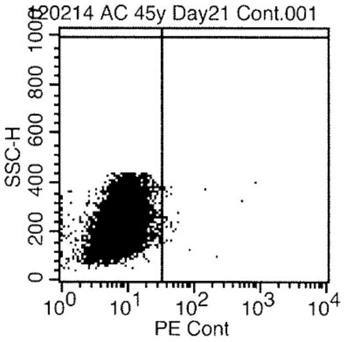

Quad	Events	% Gated
UL	0	0.00
UR	0	0.00
LL	9886	99.70
LR	30	0.30

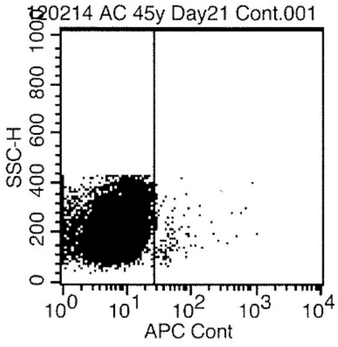

Quad	Events	% Gated
UL	0	0.00
UR	0	0.00
LL	11003	98.97
LR	114	1.03

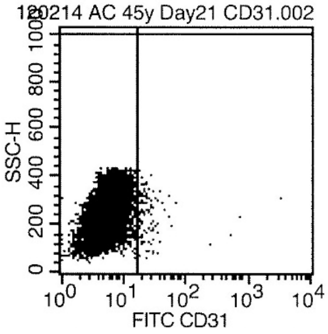

Quad	Events	% Gated
UL	0	0.00
UR	0	0.00
LL	8537	98.79
LR	105	1.21

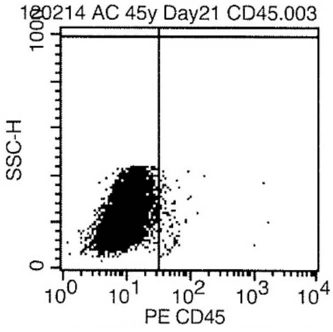

Quad	Events	% Gated
UL	0	0.00
UR	0	0.00
LL	8708	99.04
LR	84	0.96

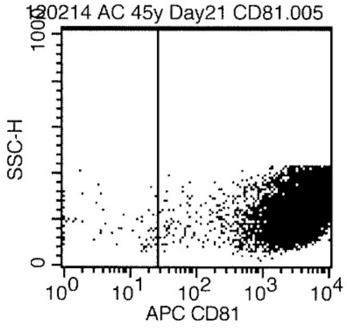

Quad	Events	% Gated
UL	0	0.00
UR	0	0.00
LL	61	0.59
LR	10312	99.41

Case 2

Triple-layered sheets (2)

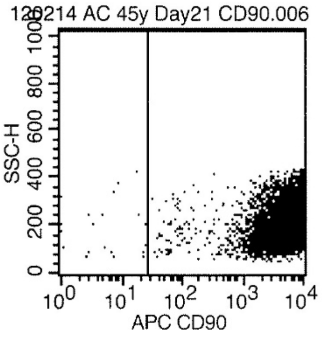

Quad	Events	% Gated
UL	0	0.00
UR	0	0.00
LL	20	0.20
LR	10177	99.80

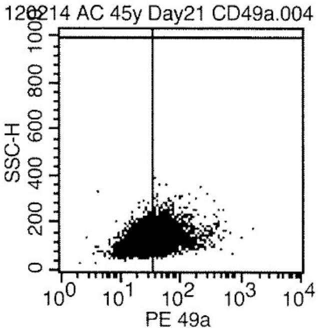

Quad	Events	% Gated
UL	0	0.00
UR	0	0.00
LL	5091	53.33
LR	4456	46.67

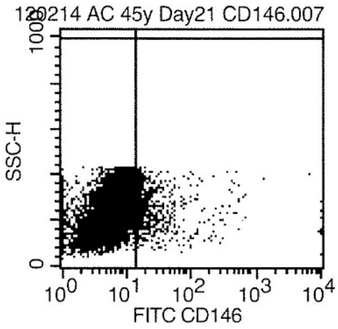

Quad	Events	% Gated
UL	0	0.00
UR	0	0.00
LL	10892	94.52
LR	632	5.48

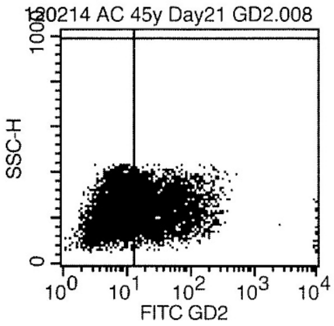

Quad	Events	% Gated
UL	0	0.00
UR	0	0.00
LL	5865	66.41
LR	2966	33.59

Case 3
Single-layered sheets (1)

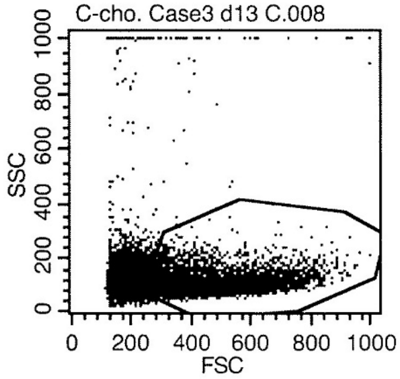

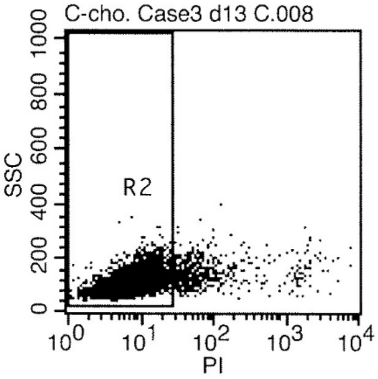

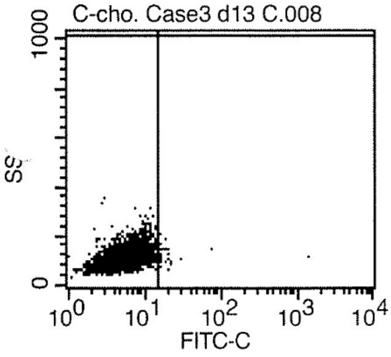

Quad	Events	% Gated	%
UL	0	0.00	
UR	0	0.00	
LL	3368	99.03	
LR	33	0.97	

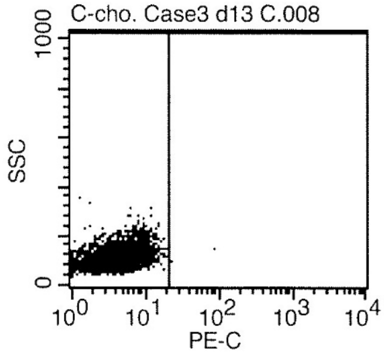

Quad	Events	% Gated	%
UL	0	0.00	
UR	0	0.00	
LL	3399	99.94	
LR	2	0.06	

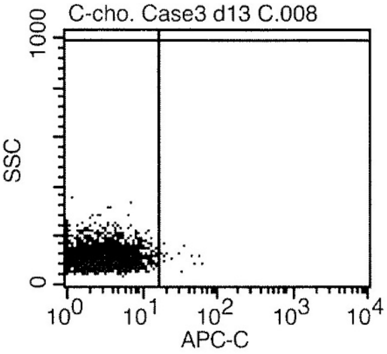

Quad	Events	% Gated	%
UL	0	0.00	
UR	0	0.00	
LL	3388	99.62	
LR	13	0.38	

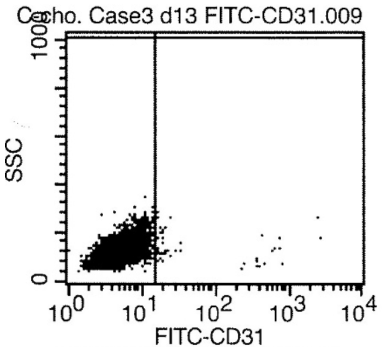

Quad	Events	% Gated	%
UL	0	0.00	
UR	0	0.00	
LL	3853	98.59	
LR	55	1.41	

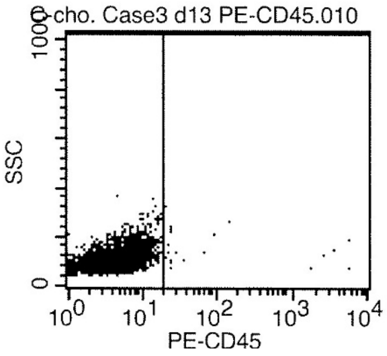

Quad	Events	% Gated	%
UL	0	0.00	
UR	0	0.00	
LL	3646	99.32	
LR	25	0.68	

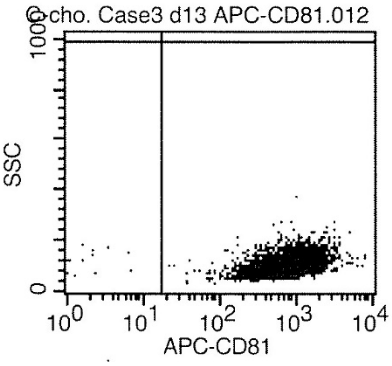

Quad	Events	% Gated
UL	0	0.00
UR	0	0.00
LL	12	0.35
LR	3441	99.65

Case 3
Single-layered sheets (2)

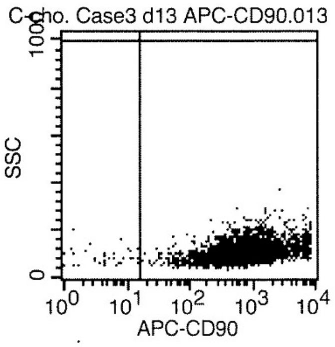

Quad	Events	% Gated
UL	0	0.00
UR	0	0.00
LL	34	0.79
LR	4272	99.21

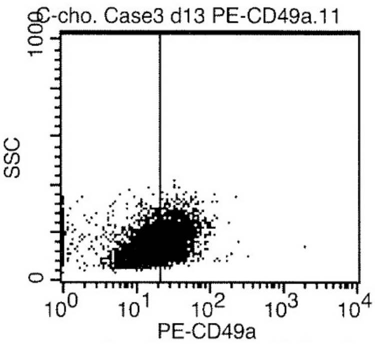

Quad	Events	% Gated
UL	0	0.00
UR	0	0.00
LL	3346	56.47
LR	2579	43.53

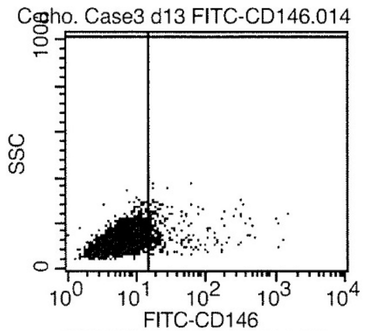

Quad	Events	% Gated
UL	0	0.00
UR	0	0.00
LL	4145	94.74
LR	230	5.26

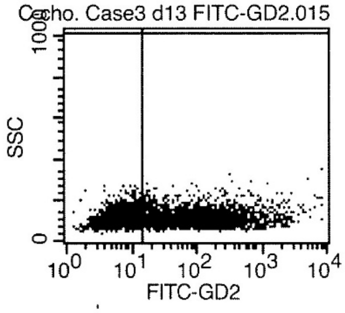

Quad	Events	% Gated
UL	0	0.00
UR	0	0.00
LL	2249	52.89
LR	2003	47.11

Case 3
Triple-layered sheets (1)

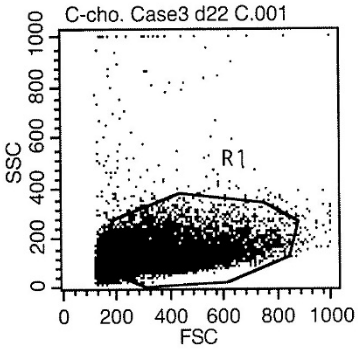

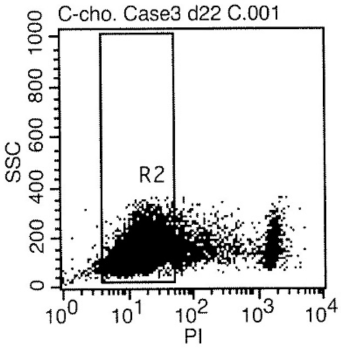

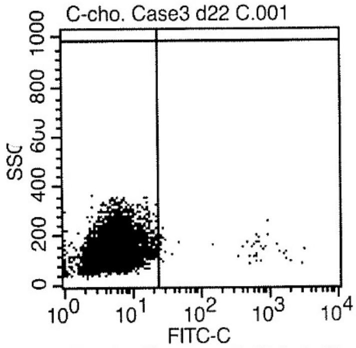

Quad	Events	% Gated	%
UL	0	0.00	
UR	0	0.00	
LL	6717	99.28	
LR	49	0.72	

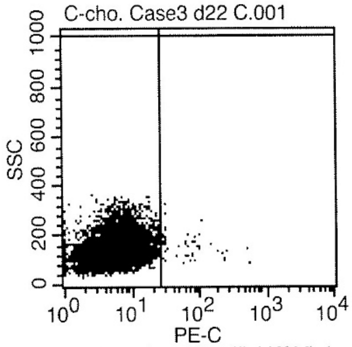

Quad	Events	% Gated
UL	0	0.00
UR	0	0.00
LL	6706	99.11
LR	60	0.89

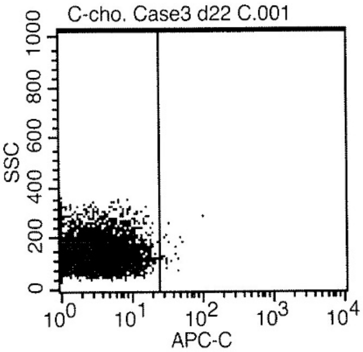

Quad	Events	% Gated
UL	0	0.00
UR	0	0.00
LL	6741	99.63
LR	25	0.37

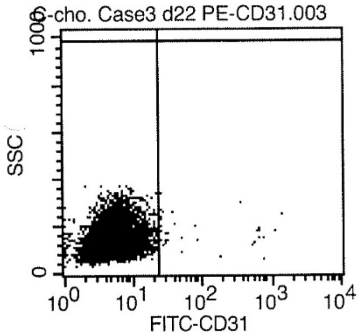

Quad	Events	% Gated	%
UL	0	0.00	
UR	0	0.00	
LL	6813	99.53	
LR	32	0.47	

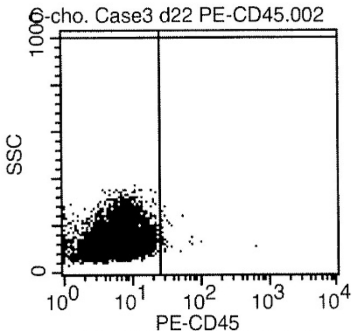

Quad	Events	% Gated	%
UL	0	0.00	
UR	0	0.00	
LL	6677	99.52	
LR	32	0.48	

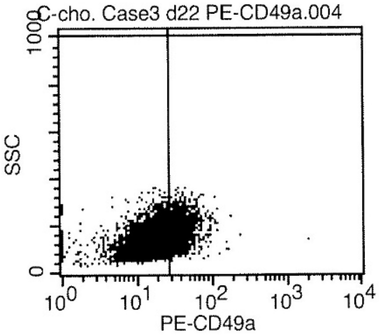

Quad	Events	% Gated
UL	0	0.00
UR	0	0.00
LL	5167	75.27
LR	1698	24.73

Case 3
Triple-layered sheets (2)

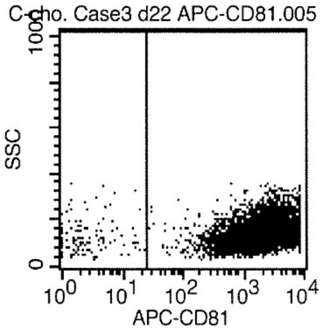

Quad	Events	% Gated
UL	0	0.00
UR	0	0.00
LL	125	1.88
LR	6517	98.12

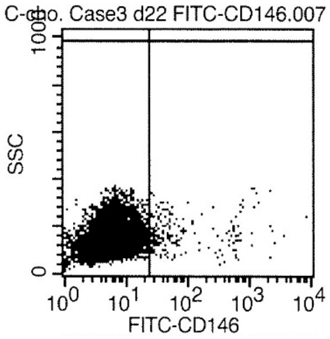

Quad	Events	% Gated
UL	0	0.00
UR	0	0.00
LL	6156	94.94
LR	328	5.06

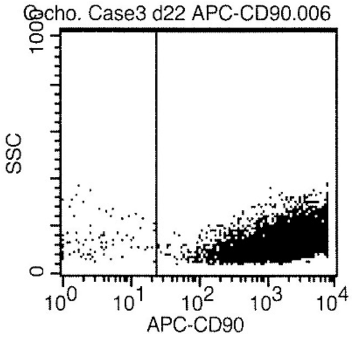

Quad	Events	% Gated
UL	0	0.00
UR	0	0.00
LL	82	1.24
LR	6552	98.76

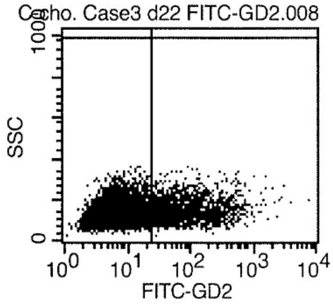

Quad	Events	% Gated
UL	0	0.00
UR	0	0.00
LL	4548	71.83
LR	1784	28.17

Case 4
Single-layered sheets (1)

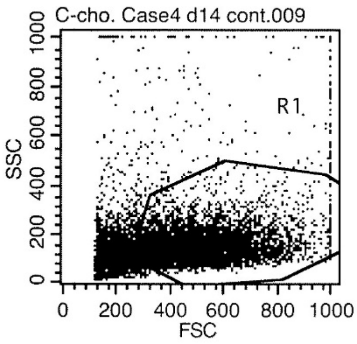

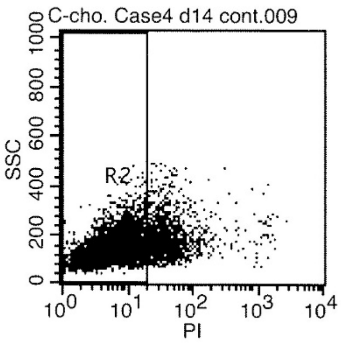

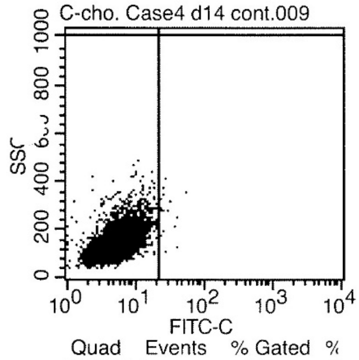

Quad	Events	% Gated	%
UL	0	0.00	
UR	0	0.00	
LL	4903	99.61	
LR	19	0.39	

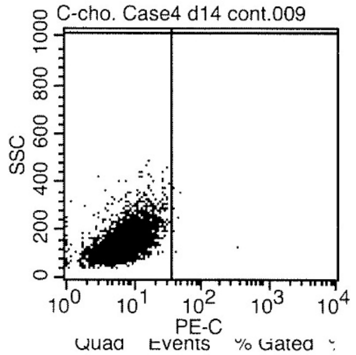

Quad	Events	% Gated	%
UL	0	0.00	
UR	0	0.00	
LL	4912	99.80	
LR	10	0.20	

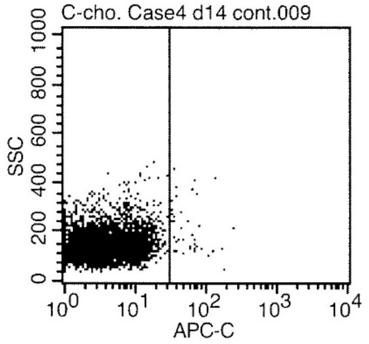

Quad	Events	% Gated	%
UL	0	0.00	
UR	0	0.00	
LL	4880	99.15	
LR	42	0.85	

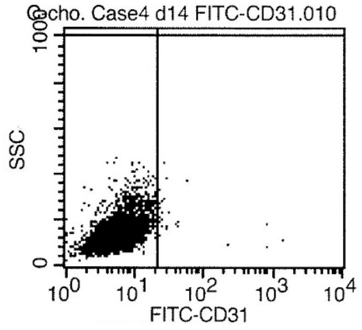

Quad	Events	% Gated	%
UL	0	0.00	
UR	0	0.00	
LL	5245	99.53	
LR	25	0.47	

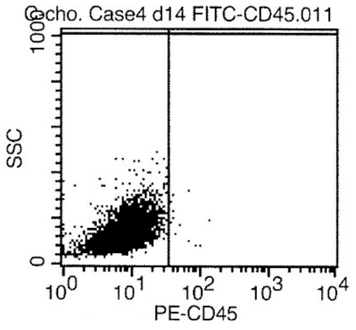

Quad	Events	% Gated	%
UL	0	0.00	
UR	0	0.00	
LL	5217	99.75	
LR	13	0.25	

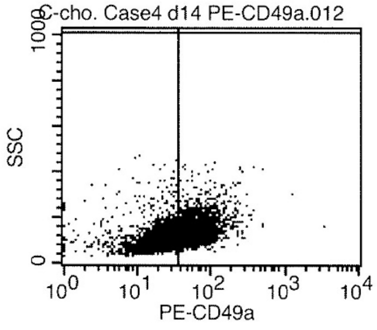

Quad	Events	% Gated	%
UL	0	0.00	
UR	0	0.00	
LL	3047	53.07	
LR	2694	46.93	

Case 4
Single-layered sheets (2)

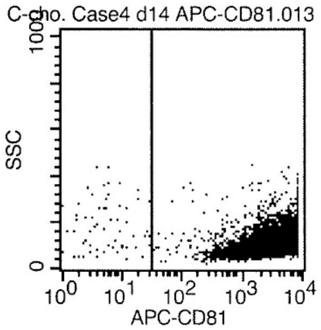

Quad	Events	% Gated
UL	0	0.00
UR	0	0.00
LL	60	1.10
LR	5416	98.90

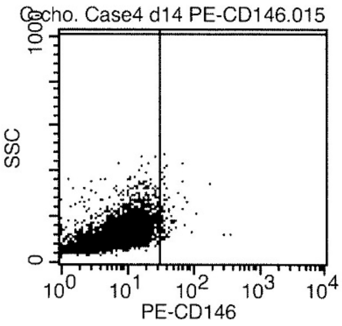

Quad	Events	% Gated
UL	0	0.00
UR	0	0.00
LL	5217	93.54
LR	360	6.46

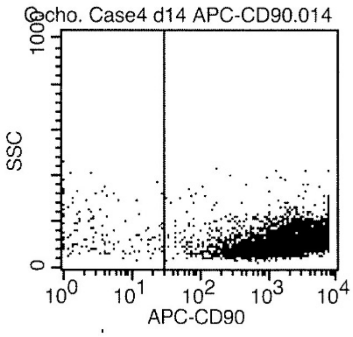

Quad	Events	% Gated
UL	0	0.00
UR	0	0.00
LL	130	2.73
LR	4637	97.27

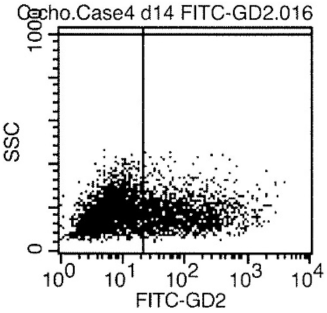

Quad	Events	% Gated
UL	0	0.00
UR	0	0.00
LL	7062	62.75
LR	4193	37.25

Case 4
Triple-layered sheets (1)

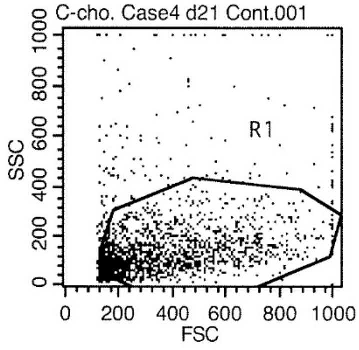

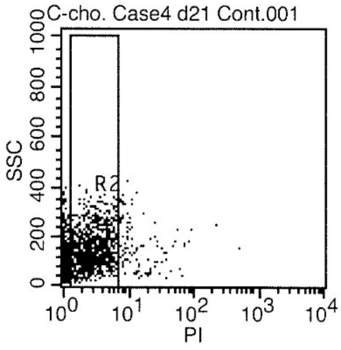

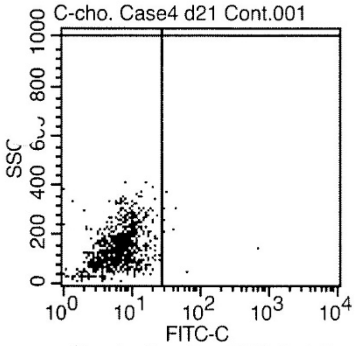

Quad	Events	% Gated
UL	0	0.00
UR	0	0.00
LL	899	99.01
LR	9	0.99

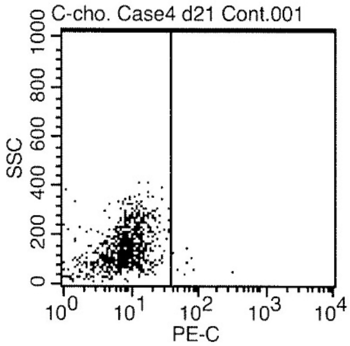

Quad	Events	% Gated
UL	0	0.00
UR	0	0.00
LL	830	99.05
LR	8	0.95

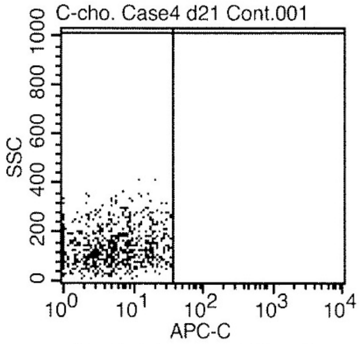

Quad	Events	% Gated
UL	0	0.00
UR	0	0.00
LL	786	99.87
LR	1	0.13

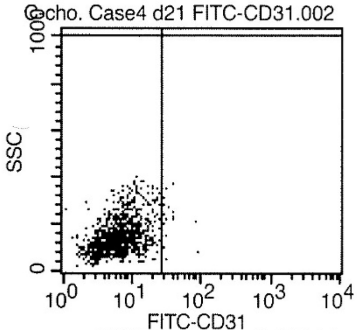

Quad	Events	% Gated
UL	0	0.00
UR	0	0.00
LL	925	98.20
LR	17	1.80

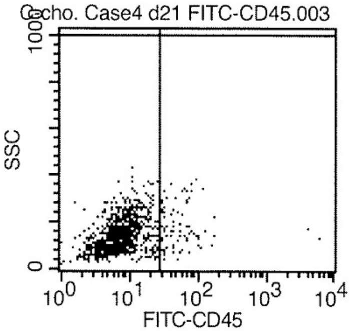

Quad	Events	% Gated
UL	0	0.00
UR	0	0.00
LL	864	95.15
LR	44	4.85

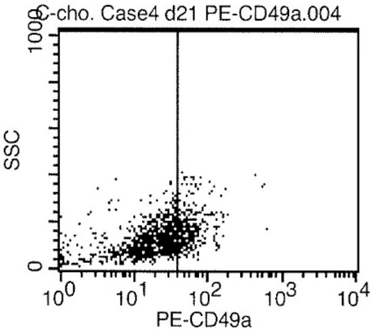

Quad	Events	% Gated
UL	0	0.00
UR	0	0.00
LL	909	75.50
LR	295	24.50

Case 4
Triple-layered sheets (2)

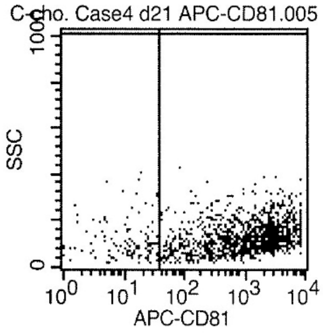

Quad	Events	% Gated
UL	0	0.00
UR	0	0.00
LL	121	9.88
LR	1104	90.12

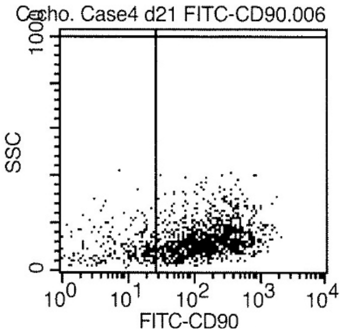

Quad	Events	% Gated
UL	0	0.00
UR	0	0.00
LL	226	16.52
LR	1142	83.48

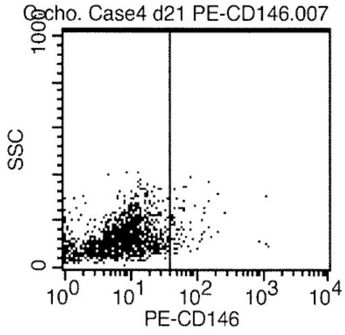

Quad	Events	% Gated
UL	0	0.00
UR	0	0.00
LL	1257	94.09
LR	79	5.91

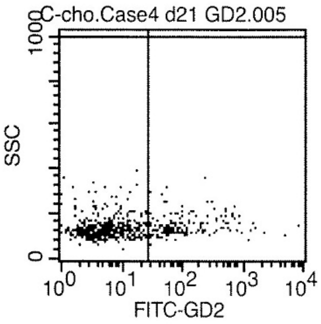

Quad	Events	% Gated
UL	0	0.00
UR	0	0.00
LL	462	73.68
LR	165	26.32

Case 5
Single-layered sheets (1)

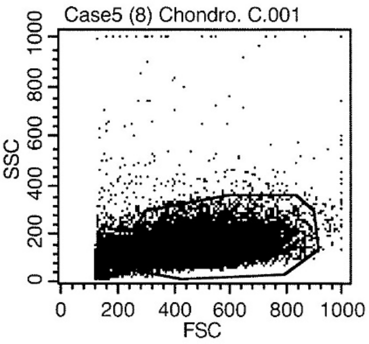

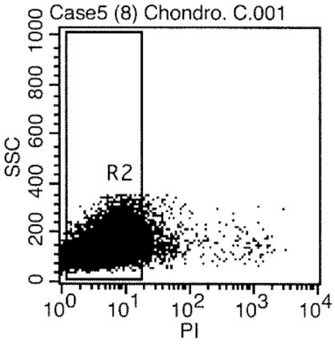

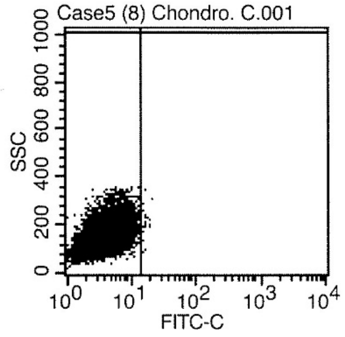

Quad	Events	% Gated
UL	0	0.00
UR	0	0.00
LL	14632	99.78
LR	32	0.22

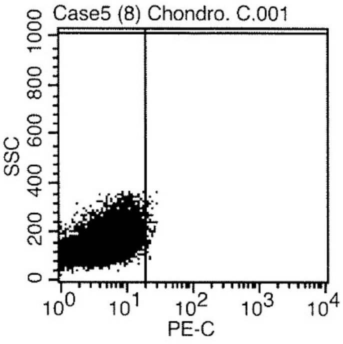

Quad	Events	% Gated
UL	0	0.00
UR	0	0.00
LL	14605	99.60
LR	59	0.40

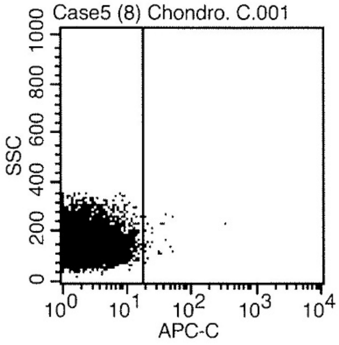

Quad	Events	% Gated
UL	0	0.00
UR	0	0.00
LL	14638	99.82
LR	26	0.18

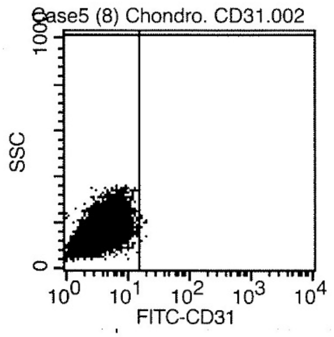

Quad	Events	% Gated
UL	0	0.00
UR	0	0.00
LL	13012	99.91
LR	12	0.09

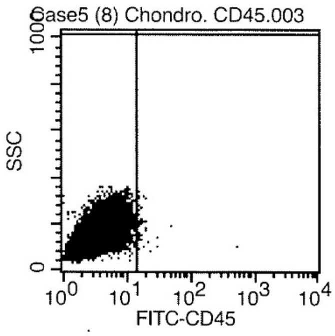

Quad	Events	% Gated
UL	0	0.00
UR	0	0.00
LL	12648	99.74
LR	33	0.26

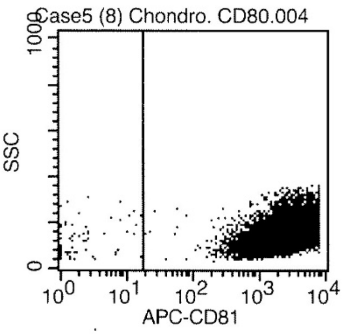

Quad	Events	% Gated
UL	0	0.00
UR	0	0.00
LL	66	0.49
LR	13533	99.51

Case 5
Single-layered sheets (2)

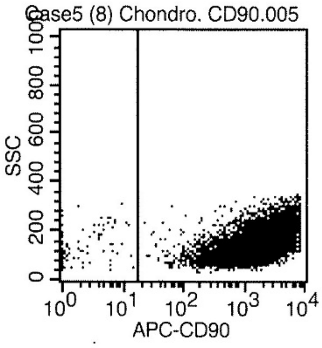

Quad	Events	% Gated
UL	0	0.00
UR	0	0.00
LL	82	0.76
LR	10753	99.24

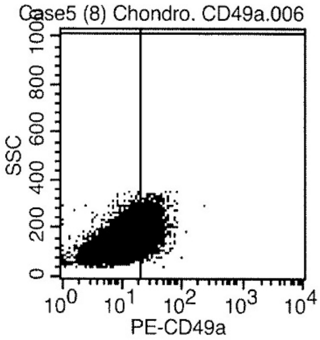

Quad	Events	% Gated
UL	0	0.00
UR	0	0.00
LL	9882	81.87
LR	2189	18.13

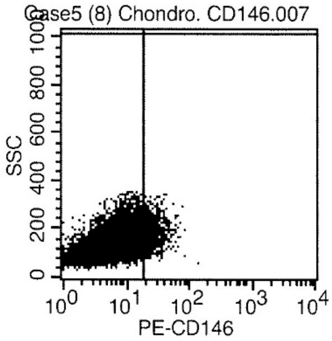

Quad	Events	% Gated
UL	0	0.00
UR	0	0.00
LL	11081	94.06
LR	700	5.94

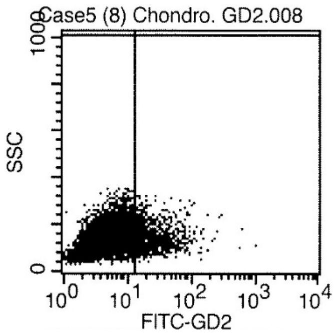

Quad	Events	% Gated
UL	0	0.00
UR	0	0.00
LL	12559	92.24
LR	1056	7.76

Case 5
Triple-layered sheets (1)

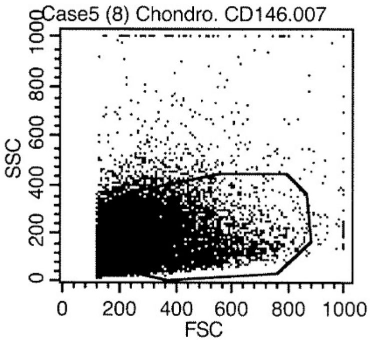

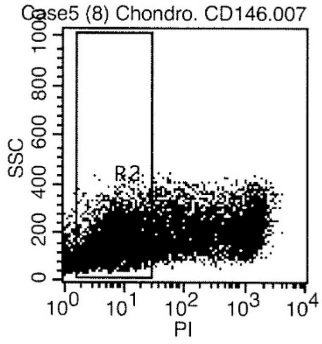

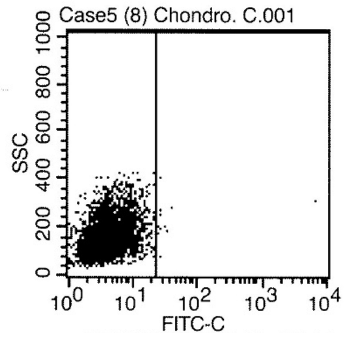

Quad	Events	% Gated
UL	0	0.00
UR	0	0.00
LL	3207	99.60
LR	13	0.40

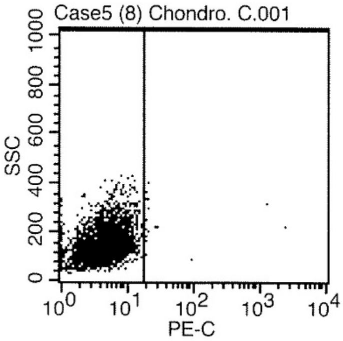

Quad	Events	% Gated
UL	0	0.00
UR	0	0.00
LL	2173	99.04
LR	21	0.96

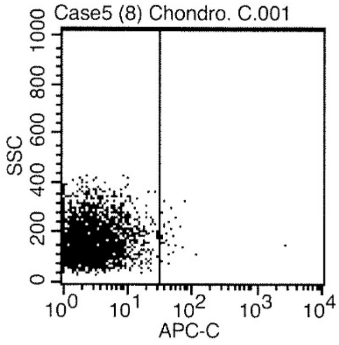

Quad	Events	% Gated
UL	0	0.00
UR	0	0.00
LL	3191	99.10
LR	29	0.90

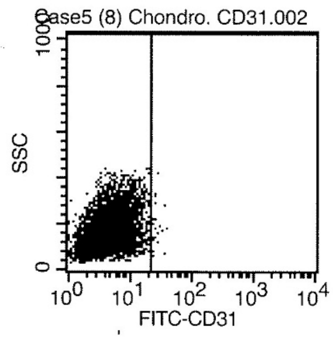

Quad	Events	% Gated
UL	0	0.00
UR	0	0.00
LL	5330	99.57
LR	23	0.43

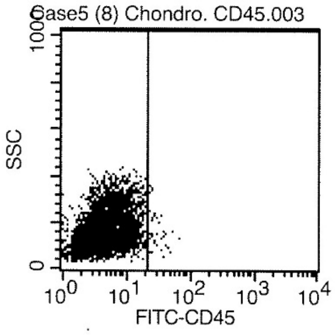

Quad	Events	% Gated
UL	0	0.00
UR	0	0.00
LL	4905	99.25
LR	37	0.75

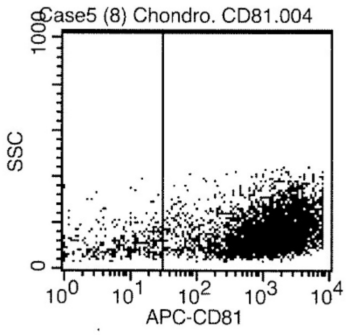

Quad	Events	% Gated
UL	0	0.00
UR	0	0.00
LL	340	6.96
LR	4546	93.04

Case 5
Triple-layered sheets (2)

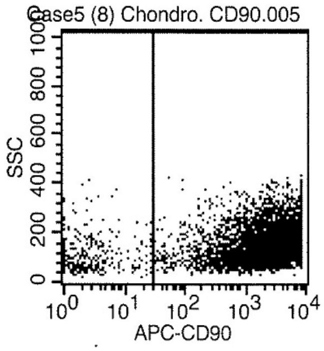

Quad	Events	% Gated
UL	0	0.00
UR	0	0.00
LL	324	6.66
LR	4539	93.34

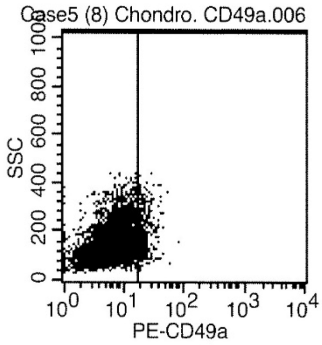

Quad	Events	% Gated
UL	0	0.00
UR	0	0.00
LL	4266	92.80
LR	331	7.20

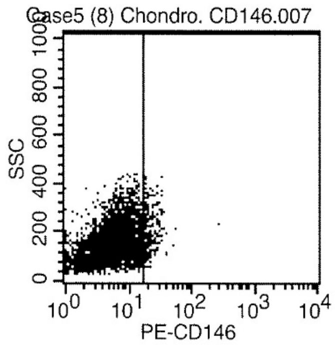

Quad	Events	% Gated
UL	0	0.00
UR	0	0.00
LL	3825	94.37
LR	228	5.63

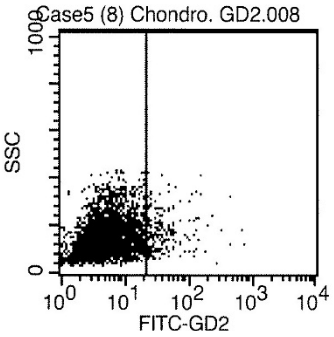

Quad	Events	% Gated
UL	0	0.00
UR	0	0.00
LL	3411	94.33
LR	205	5.67

Case 6
Single-layered sheets (1)

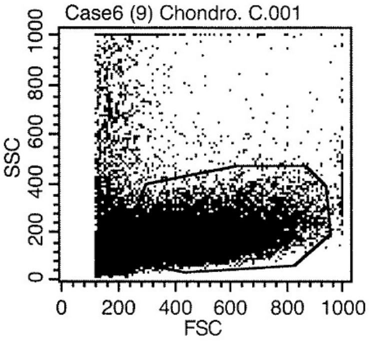

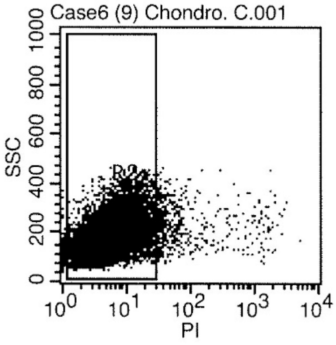

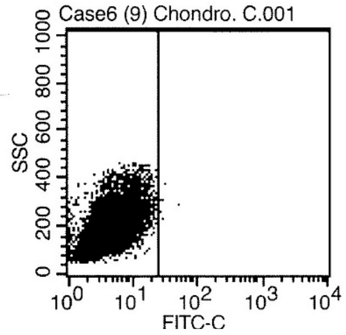

Quad	Events	% Gated
UL	0	0.00
UR	0	0.00
LL	12606	99.94
LR	8	0.06

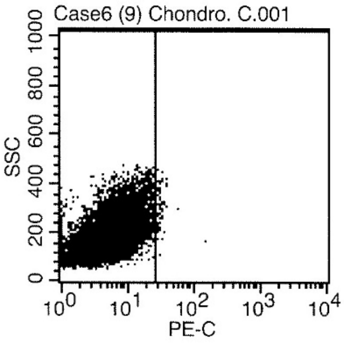

Quad	Events	% Gated
UL	0	0.00
UR	0	0.00
LL	12535	99.37
LR	79	0.63

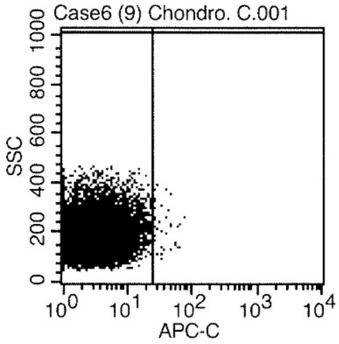

Quad	Events	% Gated
UL	0	0.00
UR	0	0.00
LL	12569	99.64
LR	45	0.36

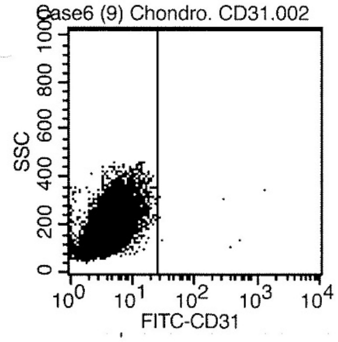

Quad	Events	% Gated
UL	0	0.00
UR	0	0.00
LL	11480	99.93
LR	8	0.07

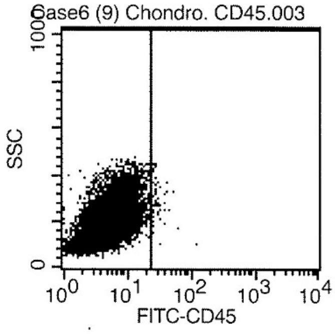

Quad	Events	% Gated
UL	0	0.00
UR	0	0.00
LL	11771	99.63
LR	44	0.37

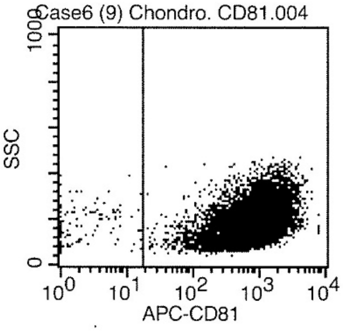

Quad	Events	% Gated
UL	0	0.00
UR	0	0.00
LL	121	1.09
LR	11030	98.91

Case 6
Single-layered sheets (2)

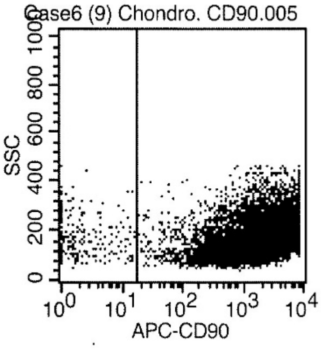

Quad	Events	% Gated
UL	0	0.00
UR	0	0.00
LL	211	1.93
LR	10739	98.07

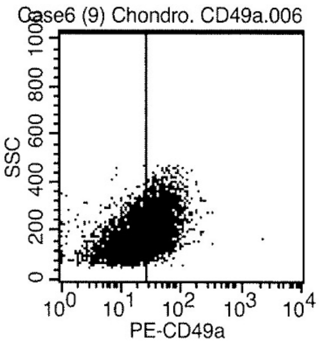

Quad	Events	% Gated
UL	0	0.00
UR	0	0.00
LL	4241	56.31
LR	3290	43.69

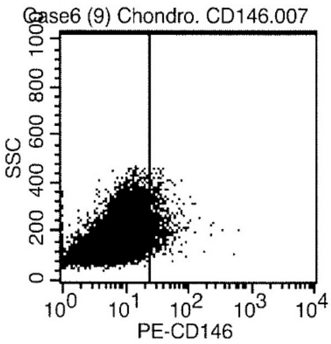

Quad	Events	% Gated
UL	0	0.00
UR	0	0.00
LL	10480	94.91
LR	562	5.09

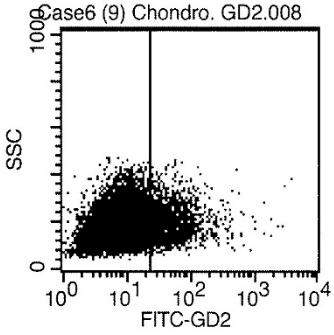

Quad	Events	% Gated
UL	0	0.00
UR	0	0.00
LL	7477	82.65
LR	1570	17.35

Case 6
Triple-layered sheets (1)

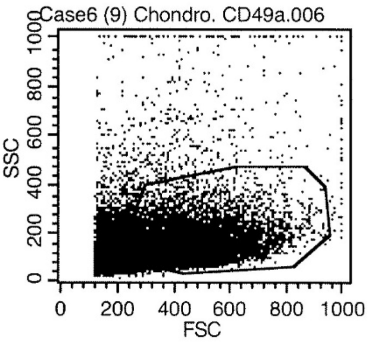

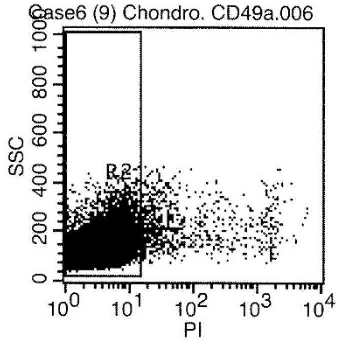

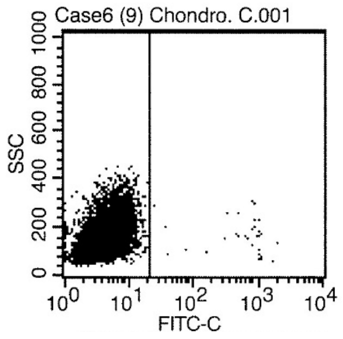

Quad	Events	% Gated
UL	0	0.00
UR	0	0.00
LL	8337	99.61
LR	33	0.39

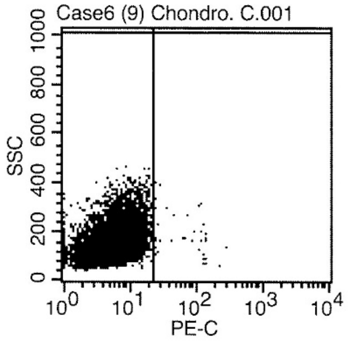

Quad	Events	% Gated
UL	0	0.00
UR	0	0.00
LL	8324	99.45
LR	46	0.55

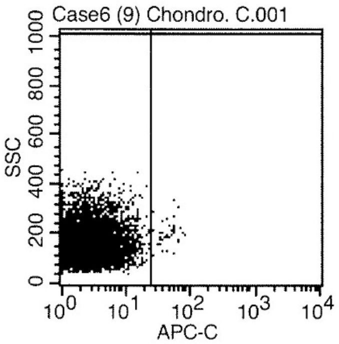

Quad	Events	% Gated
UL	0	0.00
UR	0	0.00
LL	8332	99.55
LR	38	0.45

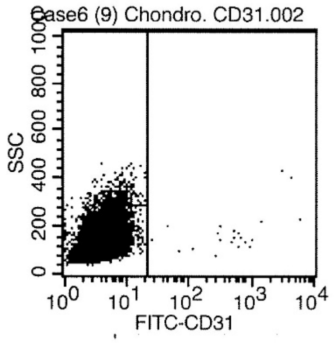

Quad	Events	% Gated
UL	0	0.00
UR	0	0.00
LL	8358	99.74
LR	22	0.26

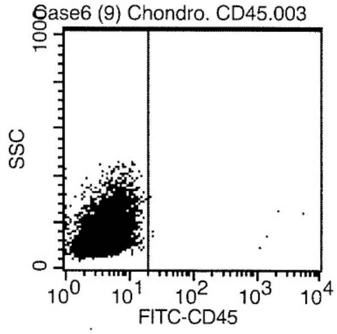

Quad	Events	% Gated
UL	0	0.00
UR	0	0.00
LL	8486	99.92
LR	7	0.08

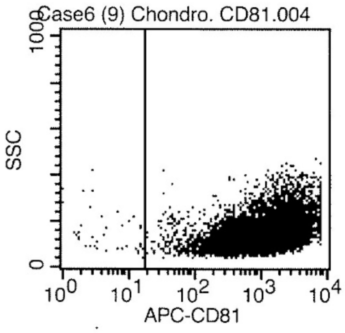

Quad	Events	% Gated
UL	0	0.00
UR	0	0.00
LL	40	0.46
LR	8644	99.54

Case 6
Triple-layered sheets (2)

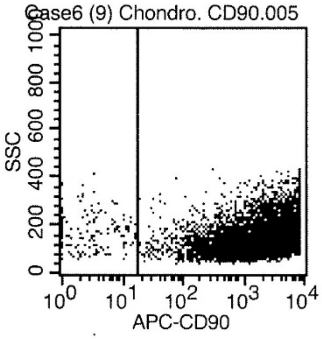

Quad	Events	% Gated
UL	0	0.00
UR	0	0.00
LL	151	1.82
LR	8141	98.18

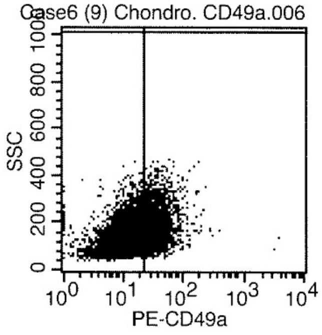

Quad	Events	% Gated
UL	0	0.00
UR	0	0.00
LL	6542	73.85
LR	2317	26.15

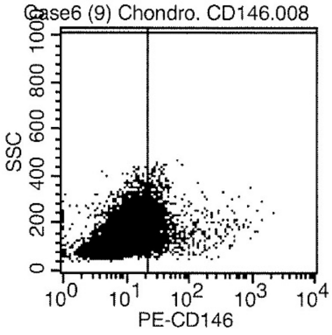

Quad	Events	% Gated
UL	0	0.00
UR	0	0.00
LL	7611	87.06
LR	1131	12.94

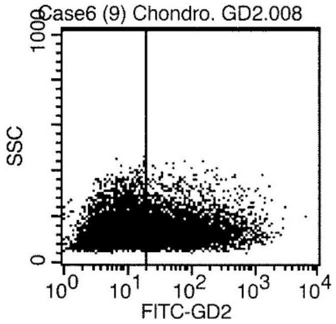

Quad	Events	% Gated
UL	0	0.00
UR	0	0.00
LL	4997	57.06
LR	3761	42.94

Case 7
Single-layered sheets (1)

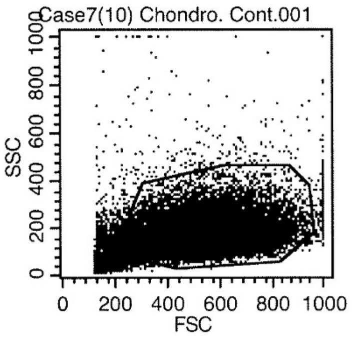

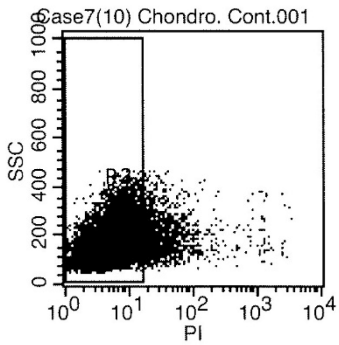

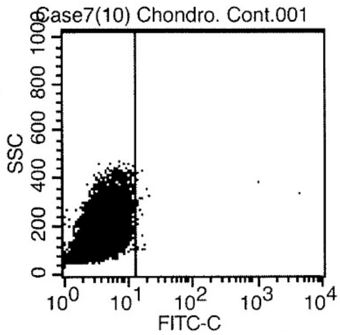

Quad	Events	% Gated
UL	0	0.00
UR	0	0.00
LL	15686	99.77
LR	36	0.23

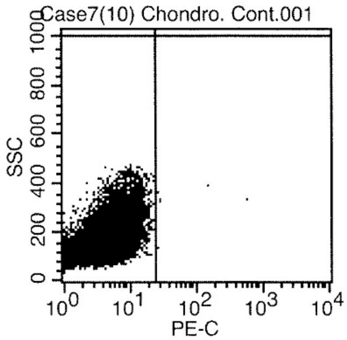

Quad	Events	% Gated
UL	0	0.00
UR	0	0.00
LL	15716	99.96
LR	6	0.04

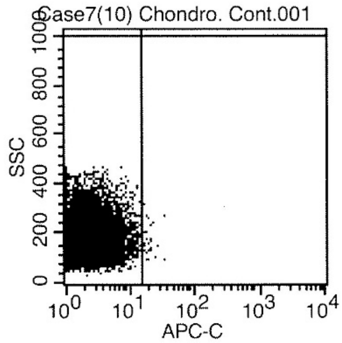

Quad	Events	% Gated
UL	0	0.00
UR	0	0.00
LL	15690	99.80
LR	32	0.20

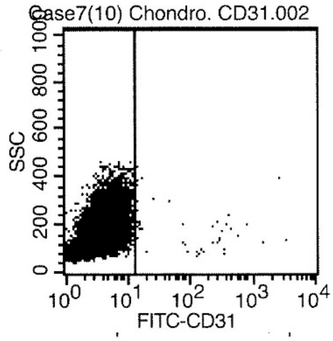

Quad	Events	% Gated
UL	0	0.00
UR	0	0.00
LL	15414	99.64
LR	56	0.36

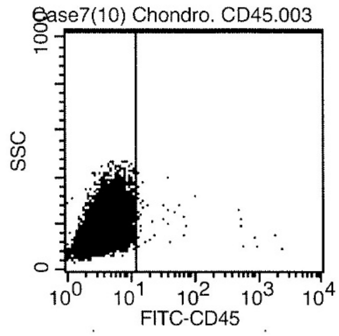

Quad	Events	% Gated
UL	0	0.00
UR	0	0.00
LL	15445	99.71
LR	45	0.29

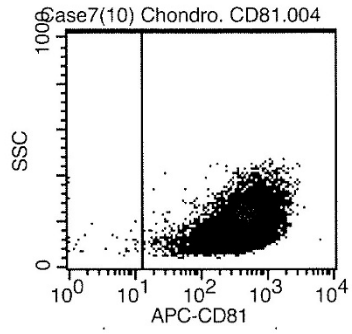

Quad	Events	% Gated
UL	0	0.00
UR	0	0.00
LL	30	0.18
LR	16325	99.82

Case 7
Single-layered sheets (2)

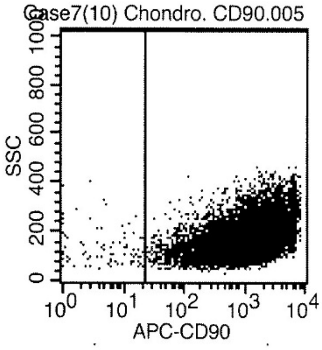

Quad	Events	% Gated
UL	0	0.00
UR	0	0.00
LL	80	0.50
LR	15847	99.50

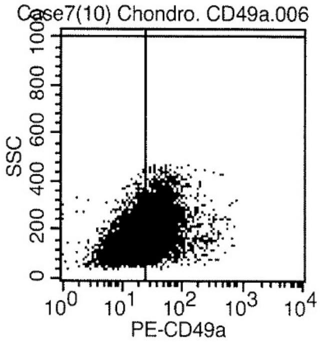

Quad	Events	% Gated
UL	0	0.00
UR	0	0.00
LL	8567	52.71
LR	7685	47.29

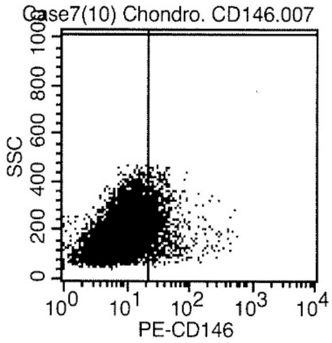

Quad	Events	% Gated
UL	0	0.00
UR	0	0.00
LL	14920	92.92
LR	1136	7.08

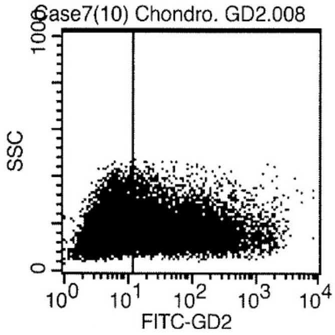

Quad	Events	% Gated
UL	0	0.00
UR	0	0.00
LL	10074	58.97
LR	7008	41.03

Case 7
Triple-layered sheets (1)

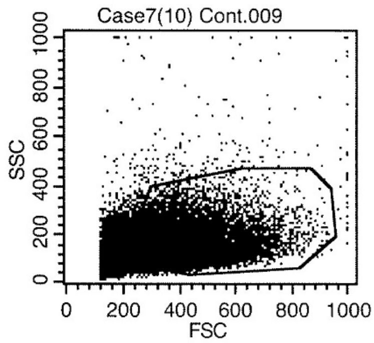

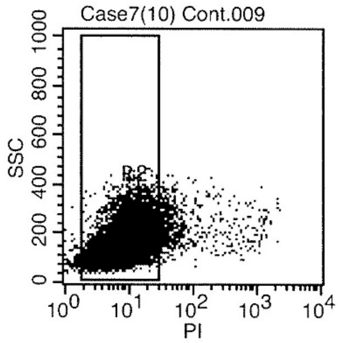

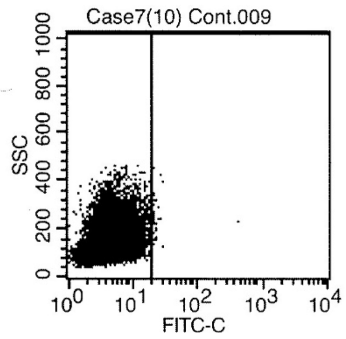

Quad	Events	% Gated
UL	0	0.00
UR	0	0.00
LL	10843	99.64
LR	39	0.36

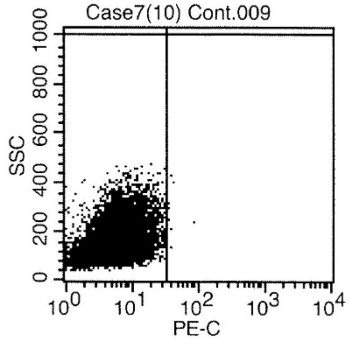

Quad	Events	% Gated
UL	0	0.00
UR	0	0.00
LL	10871	99.90
LR	11	0.10

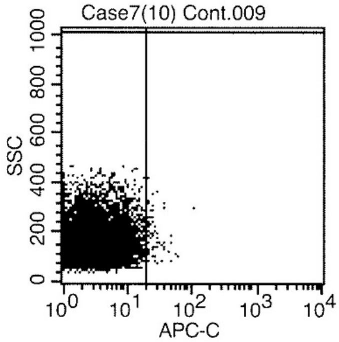

Quad	Events	% Gated
UL	0	0.00
UR	0	0.00
LL	10819	99.42
LR	63	0.58

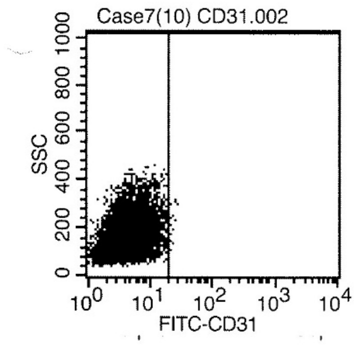

Quad	Events	% Gated
UL	0	0.00
UR	0	0.00
LL	10573	99.79
LR	22	0.21

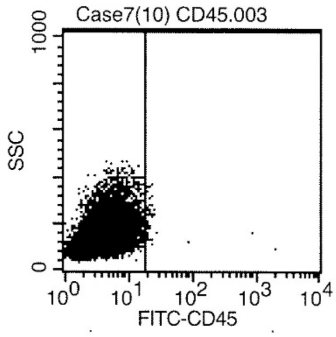

Quad	Events	% Gated
UL	0	0.00
UR	0	0.00
LL	10233	99.59
LR	42	0.41

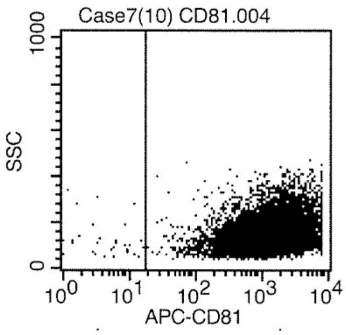

Quad	Events	% Gated
UL	0	0.00
UR	0	0.00
LL	29	0.30
LR	9795	99.70

Case 7
Triple-layered sheets (2)

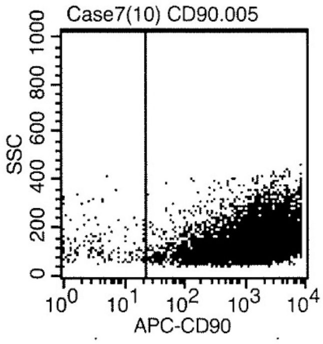

Quad	Events	% Gated
UL	0	0.00
UR	0	0.00
LL	151	1.58
LR	9400	98.42

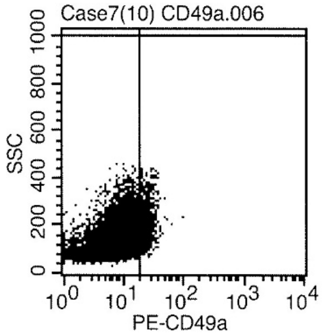

Quad	Events	% Gated
UL	0	0.00
UR	0	0.00
LL	8792	90.66
LR	906	9.34

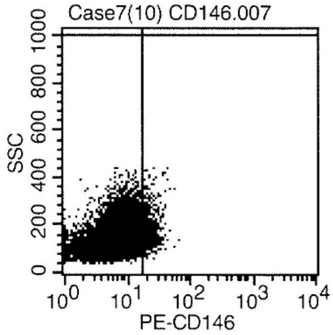

Quad	Events	% Gated
UL	0	0.00
UR	0	0.00
LL	8573	93.85
LR	562	6.15

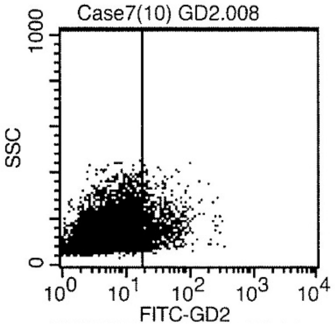

Quad	Events	% Gated
UL	0	0.00
UR	0	0.00
LL	7463	90.32
LR	800	9.68

Case 8
Single-layered sheets (1)

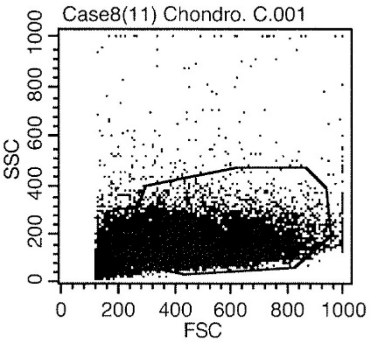

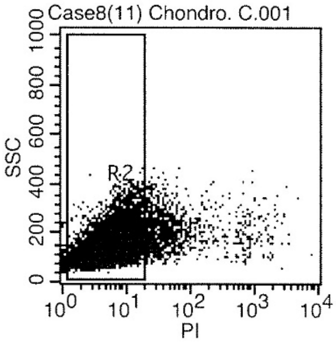

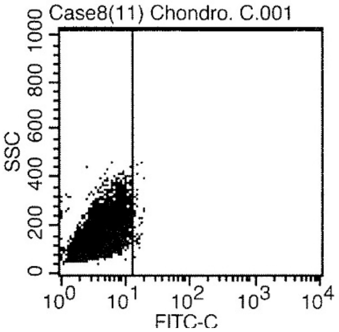

Quad	Events	% Gated
UL	0	0.00
UR	0	0.00
LL	13508	99.62
LR	52	0.38

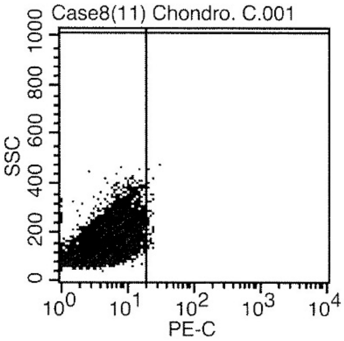

Quad	Events	% Gated
UL	0	0.00
UR	0	0.00
LL	13512	99.65
LR	48	0.35

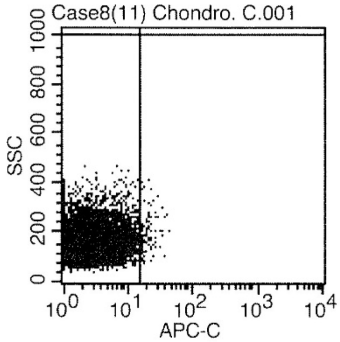

Quad	Events	% Gated
UL	0	0.00
UR	0	0.00
LL	13452	99.20
LR	108	0.80

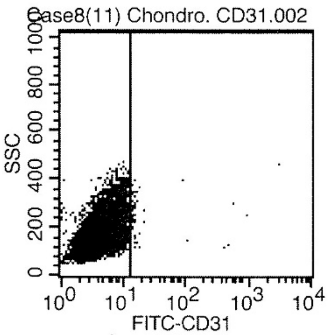

Quad	Events	% Gated
UL	0	0.00
UR	0	0.00
LL	13359	99.48
LR	70	0.52

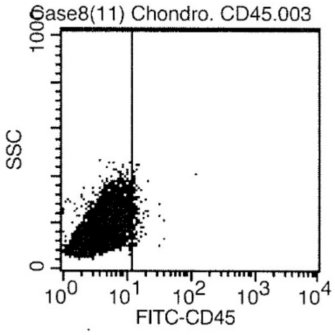

Quad	Events	% Gated
UL	0	0.00
UR	0	0.00
LL	12743	99.21
LR	102	0.79

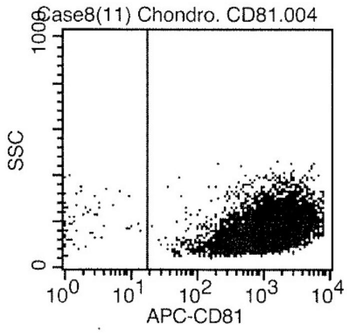

Quad	Events	% Gated
UL	0	0.00
UR	0	0.00
LL	66	0.53
LR	12500	99.47

Case 8
Single-layered sheets (2)

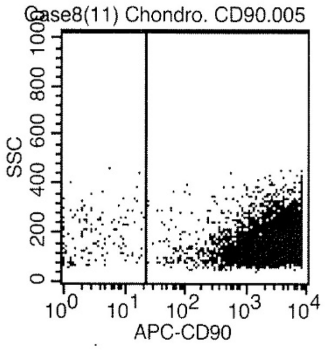

Quad	Events	% Gated
UL	0	0.00
UR	0	0.00
LL	137	1.04
LR	13068	98.96

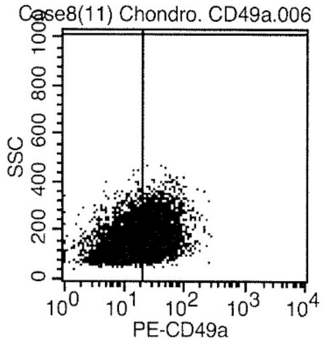

Quad	Events	% Gated
UL	0	0.00
UR	0	0.00
LL	5760	55.43
LR	4632	44.57

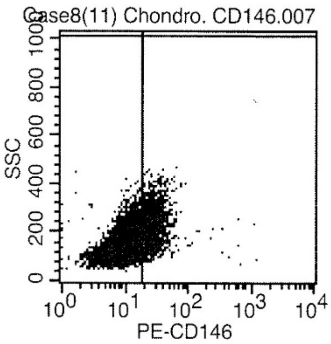

Quad	Events	% Gated
UL	0	0.00
UR	0	0.00
LL	7910	76.65
LR	2409	23.35

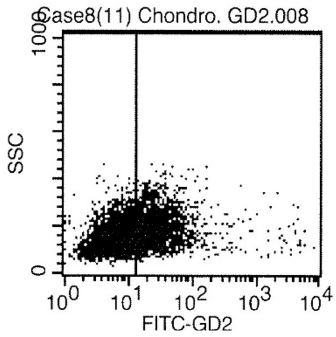

Quad	Events	% Gated
UL	0	0.00
UR	0	0.00
LL	4058	57.81
LR	2962	42.19

Case 8
Triple-layered sheets (1)

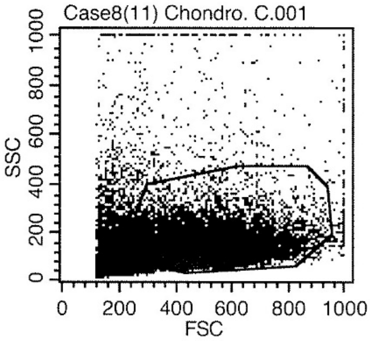

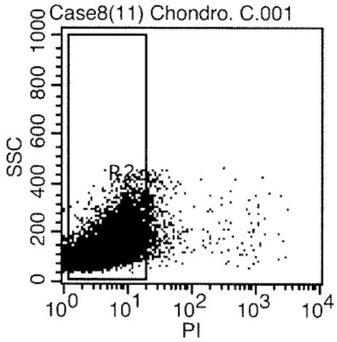

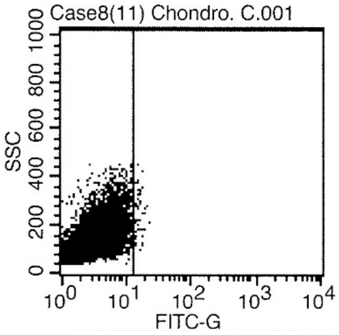

Quad	Events	% Gated
UL	0	0.00
UR	0	0.00
LL	12682	99.48
LR	66	0.52

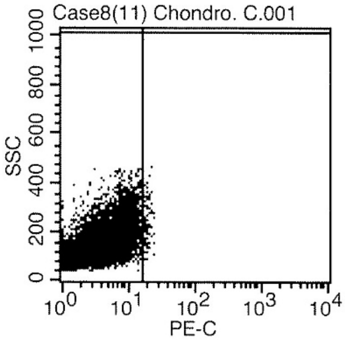

Quad	Events	% Gated
UL	0	0.00
UR	0	0.00
LL	12662	99.33
LR	86	0.67

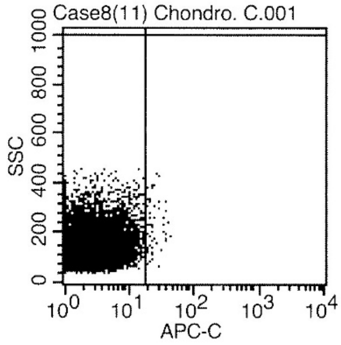

Quad	Events	% Gated
UL	0	0.00
UR	0	0.00
LL	12692	99.56
LR	56	0.44

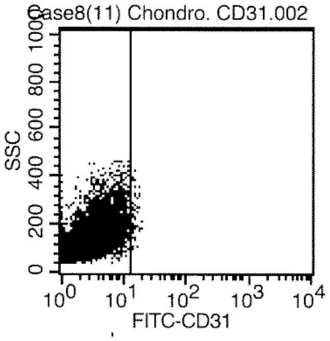

Quad	Events	% Gated
UL	0	0.00
UR	0	0.00
LL	10717	99.38
LR	67	0.62

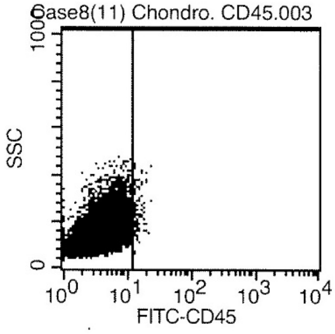

Quad	Events	% Gated
UL	0	0.00
UR	0	0.00
LL	12637	99.19
LR	103	0.81

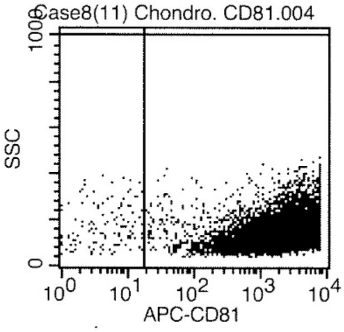

Quad	Events	% Gated
UL	0	0.00
UR	0	0.00
LL	126	1.08
LR	11576	98.92

Case 8
Triple-layered sheets (2)

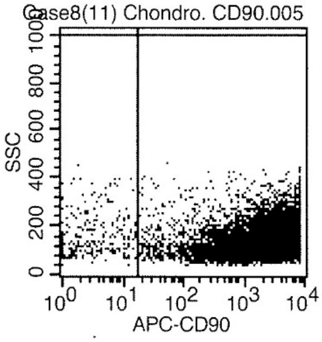

Quad	Events	% Gated
UL	0	0.00
UR	0	0.00
LL	252	2.02
LR	12232	97.98

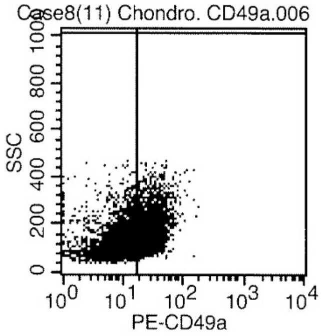

Quad	Events	% Gated
UL	0	0.00
UR	0	0.00
LL	3780	54.09
LR	3208	45.91

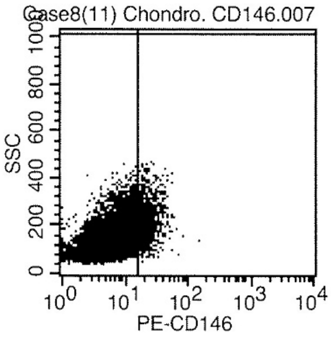

Quad	Events	% Gated
UL	0	0.00
UR	0	0.00
LL	11279	91.83
LR	1003	8.17

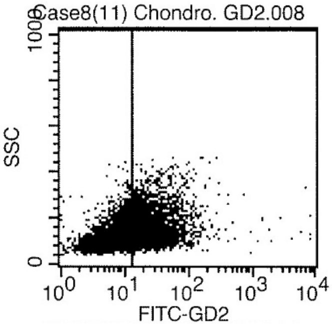

Quad	Events	% Gated
UL	0	0.00
UR	0	0.00
LL	3968	60.22
LR	2621	39.78

Clinical study for knee joint treatment using cell sheets:

Study protocol

Department of Orthopaedic Surgery, Surgical Science,
Tokai University School of Medicine

Department of Regenerative Medicine,
Tokai University School of Medicine

Department of Orthopaedic Surgery,
Tokai University Hospital

Cell Processing Center, Tokai University Hospital

Research Representative
Masato Sato

Department of Orthopaedic Surgery, Surgical Science,
Tokai University School of Medicine
143 Shimokasuya Isehara-shi, Kanagawa-ken 259-1193 Japan
0463-93-1121
0463-96-4404
sato-m@is.icc.u-tokai.ac.jp

Revised June 24, 2011

Confidentiality

This clinical study protocol is to be considered confidential information only to be shared with the review board; Clinical/Principal investigator, co-investigators, and research collaborators of the clinical study; the Dean of the Tokyo University School of Medicine; the Chairman of the Tokai University Hospital, and various departments of the Tokai University School of Medicine and Tokyo University Hospital involved in this clinical study.

Summary

１．　Objectives

This study aims to transplant cell sheets fabricated on temperature-reactive culture dishes using cells isolated from intra-articular tissues onto chondral injury sites of patients with knee joint cartilage injuries. It aims to objectively assess the safety of this new treatment modality as the primary endpoint, as well as to collect data related to efficacy by conducting clinical assessments.

２．　Subject of the study

Knee joint cartilage injury caused by trauma or degeneration.

３．　Study methods

A small sample of synovium and cartilage is harvested at the preoperative arthroscopy for diagnostic purposes. Harvested tissues are transported to the Cell Processing Center (CPC) where cells will be isolated and seeded onto a temperature-reactive culture dish on which the cell sheet will be fabricated. The cell sheet is transplanted after debriding the damaged tissue and washing of the chondral injury site. Postoperative assessment will be based on the clinical evaluation criteria. Furthermore, simple radiography, magnetic resonance imaging (MRI), arthroscopy, laser-induced photoacoustic method, and pathological testing by biopsy will be conducted according to the postoperative protocol to evaluate the postoperative regenerative state of the cartilage.

４．　Study period and target enrollment*

Three years from study approval.

10 patients.

*This clinical study may be completed without achieving the target enrollment if the assessment of safety, the primary endpoint, has been determined to be sufficiently achieved.

５．　Participating institutions

Department of Orthopaedic Surgery, Surgical Science, Tokai University School of Medicine

Department of Regenerative Medicine, Tokai University School of Medicine

Department of Orthopaedic Surgery, Tokai University School of Medicine

Cell Processing Center, Tokai University School of Medicine

Table of Contents

１. Objectives • 5
２. General information • 5
３. Background • 8
４. Ethical considerations • 9
５. Patient selection • 11
６. Clinical study period and target enrollment • • • • • • • • • • • • • • • • 12
７. Enrollment • 13
８. Study methods • 13
９. Endpoints and assessment schedule • • • • • • • • • • • • • • • • • • • 15
１０. Expected adverse events • 16
１１. Handling of adverse events • 16
１２. Medical fees and compensation • 18
１３. Statistical considerations • 18
１４. Publication of study results • 18
１５. Handling of test specimens after completion of the study • • • • • • • • • • • 19
１６. References • 19

１． Objectives

Joint cartilage treatment with cell sheets, which have validated treatment efficacy for knee cartilage injury that cannot be expected to repair spontaneously based on animal testing, will be applied to knee joint cartilage injury patients. Cells isolated from intra-articular tissues will be cultured on a temperature-reactive culture dish to fabricate cell sheets, which will be transplanted onto the site of the cartilage injury. This study seeks to objectively evaluate the safety of this new treatment modality as the primary endpoint. Furthermore, various clinical tests will be performed to collect data related to efficacy.

２． General information

１）Principal Investigator of the Clinical Study:
Masato Sato
Professor, Department of Orthopaedic Surgery, Surgical Science, Tokai University School of Medicine
Head, Cell Processing Center, Tokai University School of Medicine
(Department of Orthopaedic Surgery, Tokai University Hospital)
Management of the study.

２）Co-investigators:
Genya Mitani, Lecturer
Department of Orthopaedic Surgery, Surgical Science, Tokai University School of Medicine
(Department of Orthopaedic Surgery, Tokai University Hospital)
Clinical diagnosis, transplant, and postoperative evaluation.

Toshiharu Tsukuna, Lecturer,
Department of Orthopaedic Surgery, Surgical Science, Tokai University School of Medicine
(Department of Orthopaedic Surgery, Tokai University Hospital)
Clinical diagnosis, transplant, and postoperative evaluation.

Motonori Takagaki, Assistant Professor
Department of Orthopaedic Surgery, Surgical Science, Tokai University School of Medicine
(Department of Orthopaedic Surgery, Tokai University Hospital)
Clinical diagnosis, transplant, and postoperative evaluation.

Goro Ebihara, Assistant Professor
Department of Orthopaedic Surgery, Surgical Science, Tokai University School of Medicine
(Department of Orthopaedic Surgery, Tokai University Hospital)
Clinical diagnosis, transplant and postoperative evaluation.

Toshihiro Nagai, Assistant Professor
Department of Orthopaedic Surgery, Surgical Science, Tokai University School of Medicine
(Department of Orthopaedic Surgery, Tokai University Hospital)
Clinical diagnosis, transplant and postoperative evaluation.

Kosuke Hamahashi, Assistant Professor
Department of Orthopaedic Surgery, Surgical Science, Tokai University School of Medicine
(Department of Orthopaedic Surgery, Tokai University Hospital)
Evaluation of cell sheet properties and safety.

Kenji Serigano, Assistant Professor
Department of Orthopaedic Surgery, Surgical Science, Tokai University School of Medicine
(Department of Orthopaedic Surgery, Tokai University Hospital)
Evaluation of cell sheet properties and safety.

Satoshi Ito, Physician
Department of Orthopaedic Surgery, Surgical Science, Tokai University School of Medicine
(Department of Orthopaedic Surgery, Tokai University Hospital)
Evaluation of cell sheet properties and safety.

Taku Ukai, Physician
Department of Orthopaedic Surgery, Surgical Science, Tokai University School of Medicine
(Department of Orthopaedic Surgery, Tokai University Hospital)
Evaluation of cell sheet properties and safety.

Shunichi Kato, Professor
Department of Regenerative Medicine, Tokai University School of Medicine
Clinical study advisor
Management of the quality control of the cell sheet.

３） Research collaborators

Mami Kokubo, Special Researcher
Department of Orthopaedic Surgery, Surgical Science, Tokai University School of Medicine
Fabrication of the cell sheet and recording of the production process.
Evaluation of cell sheet properties and safety.

Yoshihiko Nakamura
Assistant Head, Cell Processing Center, Tokai University School of Medicine
Fabrication of the cell sheet and recording of the production process.
Evaluation of cell sheet properties and safety.

Taishi Mishima Research Assistant,
Department of Orthopaedic Surgery, Surgical Science, Tokai University School of Medicine
Fabrication of the cell sheet and recording of the production process.
Evaluation of cell sheet properties and safety.

４） **Clinical/Principal investigator** (Note as needed if the principal investigator is not a physician)
Same as the Principal Investigator of the Clinical Study

５） **Study institutions**
Department of Orthopaedic Surgery, Surgical Science and the Department of Regenerative Medicine, Tokai University School of Medicine
Department of Orthopaedic Surgery, Tokai University Hospital, Cell Processing Center, Tokai University School of Medicine

３．Background

１）Indicate the scientific history leading to planning this study as follows.

① Current methods for treating the condition and associated problems

Motor disorders such as osteoarthrosis (OA) do not directly jeopardize survival, and have therefore been neglected in comparison to life-threatening diseases such as cancer or heart disease. However, these disorders interfere with activities of daily living (ADL) and quality of life (QOL), and are associated with immeasurable losses in personal and social aspects of life. According to the 2008 edition of the Annual Report on the Aging Society, the elderly population, or individuals aged 65 years and above, has reached an all-time high of 27.46 million, and the proportion of the entire population (aging rate) has also reached 21.5%, marking the beginning of an unprecedented super-aging society. Within this population, the leading cause of shortened healthy life expectancy (conditions requiring medical care) is joint disease at 20.2% (2008 edition of the Comprehensive Survey of Living Conditions).

Articular cartilage defects are generally accepted to progress toward OA over 10 to 20 years if articular cartilage defects remain untreated. OA is characterized by the degeneration or disappearance of the articular cartilage and causes pain or dysfunction. OA does not directly threaten survival, but it places a significant strain on ADL and thus impacts healthy survival enormously. Overcoming OA is an important objective, as an estimated 10 million patients in Japan are affected by OA of the knee alone. Epidemiological studies have revealed that OA is both a polygenic and lifestyle-related disease that develops through interactions of both genetic and environmental factors. OA of the knee is characterized by a mild chondral injury induced by aging or load on the knee joint, which worsens to a diffuse lesion.

Mild chondral injuries are often asymptomatic. However, early treatment of even a mild chondral injury is ideal because degeneration of the extracellular matrix (ECM) of chondrocytes, which plays an important role in providing elasticity and lubrication, is observed even in cases exhibiting articular cartilage fibrillation. Furthermore, cartilage injuries that worsen into advanced cartilage injury require major, highly invasive surgical procedures including joint replacement that places immeasurably high stress on patients themselves. Currently, autologous chondrocyte transplantations are performed for mild and moderate chondral defects. However, this method has several problems such as the need to sacrifice two healthy regions, the limited number of donor sites from which transplantable cells can be harvested, and the reduced capacity of chondrocyte proliferation in the elderly; thus, the development of new treatment modalities are needed.

② Outcomes of non-clinical trials related to this study

We have conducted basic research on repair and regeneration of articular cartilage, primarily using domestic rabbits and miniature swine models. These include studies on tissue engineering to design

carriers optimized for cartilage regeneration[1], building optimal extracellular environments[2,3], repair and regeneration of allogeneic transplantation of cartilage created by tissue engineering[6,7], and cartilage repair and regeneration by cartilage cell sheet transplantation[6-8,11]. Through these studies, we have confirmed the importance of interactions between the host (recipient) and donor cells in cartilage repair and regeneration, and found the minimum amount of cartilage induction initiators (tissue-engineered cartilage) required for tissue repair and regeneration which stimulates the host cells (recipient) to promote repair[4,10]. We have reported the first effective repair and regeneration of articular cartilage with layered cartilage cell sheets fabricated on temperature-responsive culture dishes suitable for treatment of partial thickness defects of articular cartilage, which have conventionally been believed to repair poorly[6]. Furthermore, we have elucidated the properties of the layered cartilage cell sheets with high repair capacity[7,12]. Effective cartilage repair has been confirmed in a miniature swine full-thickness defect model, confirming that layered cell sheets were effective for both partial and full-thickness articular defects[13].

Overall, our studies have shown the efficacy of cell sheets to treat these concomitant types of cartilage injury that occur consistently in OA. Cell sheet engineering, an innovative Japanese technique is expected to play an important role in the treatment of OA as cartilage regenerative medicine.

1) Sato M. et al, J Biomed Mate Res A 2003; 64(2):248-256. 2) Ishihara M. et al, Biomaterials 2002; 23(3):833-840. 3) Ishihara M. et al, J Biomed Mater Res 2001; 56(4):536-544. 4) Masuoka K. et al, J Biomed Mater Res B 2005; 75(1):177-184. 5) Sato M. et al, J Biomed Mater Res B 2007; 83(1):181-188. 6) Kaneshiro K. et al, Biochem Biophys Res Commun. 2006 349(2): 723-731. 7) Kaneshiro N. et al, Eur Cell Mater 2007; 13: 87-92. 8) International Application Number: PCT/JP2006/303759 Application date: February 28, 2006. Applicant: CellSeed, Inc. Inventor: Masato Sato et al. 9) Nagai T. et al, Tissue Engineering - Part A. 2008; 14(7):1183-1193. 10) Nagai T. et al, Tissue Engineering - Part A 2008; 14(7):1225-1235. 11) Sato M. et al, Med Biol Eng Comput 2008; 46(8):735-743. 12) Mitani G. et al, BMC Biotechnology 2009; 9:17. 13) Sato M. et al, J Jpn Orthop Assoc 2008; 82(8): S930.

４. Ethical considerations

１）Compliance with ethical guidelines related to clinical studies

This clinical study is to be conducted in compliance with the ethical principles of the “Ethical guidelines for clinical studies”, “Rules and Bylaws on Clinical Studies at Hospitals affiliated with

the Tokai University School of Medicine”, “Guidance for quality and safety assurance of cell tissue derived medical devices and drugs”, “Guidelines on clinical research using human stem cells”, Appendix 1 “Evaluation index on articular cartilage regeneration” of the “Publication of next-generation medical device evaluation indices”, and this clinical study protocol.

２） Ethics review board

Approval by the medical ethics review board of the Tokai University School of Medicine is required to conduct this clinical study. The Principal Investigator of the Clinical Study shall submit the “Report of Current Status of Clinical Study” to the ethics review board annually, and comply with the decision of the review board on whether or not the study should be continued thereafter. Furthermore, the Principal Investigator shall submit the “Clinical Study Completion (Discontinuation) Report” to the review board within a month of completion or discontinuation of the study. The report shall be submitted within one week of occurrence in the event of ①, as indicated below.

The Principal Investigator shall report any of the following occurrences to the Clinical Study Ethics Review Board and shall comply with the above review board’s judgment on whether or not the study should be continued thereafter:

① Serious adverse events.
② Major modifications to the clinical study protocol.
③ Major modifications to the consent form and other documents.
④ Modifications to other documents that are subjected to review.
⑤ Other situations in which the chairman of the hospital deems that a review is required.

３） Methods for obtaining consent of participants

Free and informed written consent for enrollment as a study participant is to be obtained prior to the initiation of this clinical study using the information sheet provided by the Clinical/Principal Investigator or co-investigator physicians of the clinical study based on the following items:

① Research aspects involved in the study.
② Objectives.
③ Methods (exploratory nature of the study, patient selection criteria, etc.).
④ Scheduled period of participation.
⑤ Scheduled number of participants.
⑥ Potential clinical risks, benefits, or losses (the patient must be so informed if there are no potential clinical benefits).
⑦ Availability of other treatment modalities for patients and predicted major risks and benefits

associated with those modalities.

⑧ Treatments available to the participant in the event of health damage related to the study.

⑨ Free nature of participation in the study, such that the participant or legal representative may refuse or withdraw from the study at any time, and that the participant will not be subjected to any disadvantage or unfair treatment following refusal to participate or withdrawal from the study.

⑩ Patients and or their legal representatives will promptly be informed of any findings obtained during the study that may affect the patient's decision to continue participation in the study.

⑪ Potential conditions or reasons for the investigator to terminate the patient's participation.

⑫ Participant's confidentiality (privacy) will be protected when the clinical study results are published.

⑬ Participants will not be responsible for paying any costs associated with the study.

⑭ Names, titles, and contact information of the Principal Investigator physician and co-investigators of the clinical study.

Contacts of the medical institution for further inquiries on the clinical study and the participant's rights.

⑮ Responsibilities of the participant.

⑯ Conflicts of interest.

４） Supplying information to participants

The Principal Investigator physician of the clinical study will promptly inform participants of any findings obtained during the study that may affect the participant's decision to continue participation in the study. He/she will confirm the patient's willingness to continue his/her participation in the study, and will record the transaction and decisions in the medical records.

５） Confidentiality

Cell sheets are fabricated individually for each participant. Therefore, the Principal Investigator, co-investigators of the clinical study, and all participating physicians affiliated with the Department of Orthopaedic Surgery may have access to the identity of participants. Furthermore, clinical data (information on medical charts, pre- and postoperative testing data, imaging data) are all recorded on the electronic chart system of the institution, therefore all physicians working at the Tokai University Hospital have professional access to patient information; in other words, a system of anonymization specific to this clinical study will not be in place. However, anonymity as a general inpatient is maintained, and personal information is handled using a patient identification number. Furthermore, data will be protected by linkable anonymization following the completion of the clinical trial and clinical data will be analyzed and published in adherence with the rules of confidentiality to protect

the privacy of participants.

５．Patient selection

１）Eligible patients

Patients with knee joint cartilage injury caused by trauma or degeneration are eligible to participate in this study.

２）Selection, exclusion, and Withdrawal/Discontinuation criteria

（１）Selection criteria

Participants shall meet all the following selection criteria and have the legal capacity to provide their consent:

① Patients aged 20–60 years of either sex.

② Patients with knee osteochondral injury.

③ Patients with arthroscopy findings indicative of cartilage injury of Outerbridge classification Grade III or IV.

④ Patients with cartilage defects measuring 1.0 cm^2–4.2 cm^2 on the medial or lateral femoral condyles, which are indications for conventional bone marrow stimulation or mosaicplasty.

（２）Exclusion criteria

Patients who meet one or more of the following criteria will be excluded:

① Patients or their families that require special considerations and are ethically problematic.

② Patients with severe complications.

③ Patients with infections that pose potential problems (e.g., HBV, HCV, HIV, HTLV, FTA-ABS-positivity).

【Rationale】

［Exclusion criteria were established in consideration of the participant’s safety］

Criteria ① - ③ were determined in consideration of the safe and ethical conduction of this clinical study as well as to obtain reliable data.

（３） Withdrawal/Discontinuation criteria

Participation in the clinical study is immediately terminated in the event of any of the following

regardless of the participant’s consent:

① The participant or family wishes to withdraw from the clinical study.
② Surgery must be discontinued due to intraoperative exacerbation of the general state.
③ Intraoperative findings require changes to the scheduled surgical method.
④ Intraoperative findings indicate that the preoperative diagnosis was unsuitable.
⑤ A serious complication is induced due to an intraoperative complication.
⑥ The occurrence of serious adverse events.

６． Clinical study period and target enrollment

Period of the clinical study: Three years from approval
Target enrollment: 10 patients

【Rationale】

Approximately thirty patients annually undergo surgery for articular cartilage injury, requiring a combination of anterior cruciate ligament reconstruction or high tibial osteotomy at the Tokai University Hospital. Target enrollment was set as the number of patients deemed appropriate for the study period considering the selection criteria, and that approximately three weeks are required for the incubation period for fabrication of the cell layer and are necessary to avoid introduction of cells of other participants to the Cell Processing Center to prevent cross contamination. This clinical study shall be terminated without achieving target enrollment if the safety evaluation, the primary endpoint, is determined to be sufficiently achieved.

７． Enrollment

The Principal Investigator verifies that all candidates have met the eligibility criteria and do not satisfy any exclusion criteria to be enrolled as study participants.

８． Study methods

１） This study is a clinical study conducted in accordance with the “Guidelines on clinical research using human stem cells.”
(a clinical study, for which an application for registration has been made to the Ministry of Health, Welfare and Labor following the obtainment of approval of the Institutional Review Board, and is approved by the Minister of Health, Welfare and Labor)

２）**Methods**

Sufficient time will be expended to obtain informed consent from eligible patients and their families once necessary time is dedicated to provide detailed information on preoperative arthroscopy and on cell sheet transplantation. The clinical study is initiated after informed consent is obtained at these two time points. The degree of cartilage injury is verified on arthroscopy testing, and the cartilage required is harvested from the synovium (1 g or more) and from the unloaded portion of the femoral side of the joint (3 g or more) to fabricate the cell sheet. Harvested tissues are transported from the operating room to the Cell Processing Center for cell isolation and fabrication of the cell sheet on temperature-reactive culture dishes. The fabricated cartilage cell sheet (the final product) is then transplanted to the region consisting of the cartilage defect at the time of the scheduled surgery of the participant. Several sheets may be transplanted according to the size of the cartilage defect. If the cartilage defect is composed of defective tissue, this tissue will be debrided and the lesion washed, after which the cell sheet will be transplanted directly above the defective site so as to cover it completely. The cell sheet does not require suture to surrounding tissues. Postoperatively, safety and efficacy of the treatment modality over time is evaluated according to predetermined testing and modalities.

３）**Safety endpoints of the cell sheet**

The following evaluations will be performed to ensure the pre-transplantation safety of the cell sheet fabricated from patient-derived cells (autologous cells) prior to the submission of the specimen on the date indicated in section 5) below: cell morphology observation, endotoxin testing, mycoplasma and virus detection testing, and sterility testing. These are performed at various intervals between submission of cells to the CPC to the day of the transplantation to confirm the safety of the tissue for transplantation and to evaluate the conditions prior to fabrication at the CPC, as interim testing during fabrication, and on the day before transplantation, and to verify the conditions of the final product.

４）**Efficacy endpoints of the cell sheet**

The following tests will be conducted to evaluate the efficacy of the cell sheets fabricated in the CPC prior to submitting the specimen on the date indicated in 5) below: property testing (real-time PCR), delamination test, cell count, and cell purity (flow cytometry) testing. These tests will be performed at various intervals dating from the submission of cells to the CPC to the day of the transplantation. Testing for detailed properties other than those listed in the table below will be conducted at a later date.

５）Summary of endpoints

		Test schedule					
	Specimen name	Cartilage at time of receipt	Synovium at time of receipt	Interim control 1)	Interim control 2)	Cartilage on day before transplantation	Cartilage on day of transplantation
	Specimen submission date	Day 0	Day 0	First culture medium exchange	Layering	Day before transplantation	On the day of transplantation
Test item							
Date of receipt of results							
Mycoplasma (PCR method)							
Date of receipt of results							
Mycoplasma (culture method)							
Date of receipt of results							
Bacterial culture (aerobic)							
Date of receipt of results							
Bacterial culture (anaerobic)							
Date of receipt of results							
Sterility testing (membrane filtration)							
Date of receipt of results							
Delamination test							
Date of receipt of results							
Endotoxin (turbidimetric method)							
Date of receipt of results							
Synoviocyte surface marker test	(CD31)				%		
	(CD45)				%		
	(CD105)				%		
	(CD146)				%		
	(CD166)				%		
	(CD271)				%		
Date of receipt of results							
Chondrocyte surface marker test	(CD31)				%	%	
	(CD45)				%	%	
	(CD81)				%	%	
	(CD90)				%	%	
	(CD146)				%	%	
	(CD49a)				%	%	
	(CD2)				%	%	
	(COL2)				%	%	
	(proteoglycan)				%	%	
Date of receipt of results							
Chondrocyte sheet real-time PCR	(COL2)						
	(COL1)						
Date of receipt of results							
HBV (DNA)							
HCV (DNA)							
HIV-1 (DNA)							
HIV-1 (RNA)							
HTLV-1 (RNA)							
HTLV-1 (DNA)							
Date of receipt of results							

Note the results of the test submission date (date of test) and date of receipt of results (test result day) will be accordance with this table.

９． Postoperative testing, clinical evaluation, and schedule

１）Clinical evaluation

Clinical symptoms are observed during hospitalization and on outpatient visits. Endpoints of

clinical evaluation included the Tegner-Lysholm Knee Scoring Scale score and the Knee Injury and Osteoarthritis Outcome Score evaluated preoperatively and at one, three, six, and 12 months postoperatively.

２） Simple radiography

Cleft between articulations, conditions of the subchondral bone, and evidence of progression to arthrosis will be evaluated objectively, preoperatively and at one, three, six, and 12 months postoperatively using the Kellgren-Lawrence grading scale.

３） Magnetic resonance imaging

Changes to thickness and properties of the cartilage over time will be evaluated objectively preoperatively, and at one, three, six and 12 months postoperatively using Nelson MRI Grading and magnetic resonance observation of cartilage repair tissue (MOCART) scores.

４） Arthroscopy

Cartilage characteristics (color, hardness, smoothness) on the joint surface will be evaluated at postoperative year one using the Outerbridge classification. Additional assessments will be performed as needed in the event of pain or joint swelling.

５） Photoacoustic method

Cartilage of the transplant site and surrounding cartilage will be evaluated under arthroscopy using a unique functional diagnostic device developed by the authors, which quantifies the viscoelasticity of the articular cartilage, at postoperative year one. This evaluation method has been approved by the Institutional Review Board for Clinical Research of the Tokai University School of Medicine, and is clinically applied routinely at the Tokai University Hospital.

６） Histological evaluation

A portion of the regenerated tissue will be biopsied during the arthroscopy and will be stained with Safranin-O for an objective histological evaluation and will be assessed according to the Mankin Score and other scores.

Endpoints

① Cumulative number of serious adverse events and scores based on the clinical evaluation criteria at postoperative year one.

② Scores based on simple radiography evaluation criteria at postoperative year one.

③ Scores on MRI evaluation criteria at postoperative year one.

④ Evaluation of arthroscopy findings at postoperative year one.
⑤ Evaluation of viscoelasticity based on the photoacoustic method at postoperative year one.
⑥ Histological evaluation scores at postoperative year one.

Clinical evaluations 1) to 3) above will be continued for as long as possible postoperatively.

１０．Expected adverse events

Over 200,000 autologous cultured chondrocyte transplantations have been performed worldwide. There have been reports of postoperative infection, a common risk for surgical procedures in general, but there have been no previous reports of malignant transformation. Thickening and ossification of the transplanted periosteum is possible in procedures combining periosteal transplantation, but the present study does not use the periosteum. The transplantation of the cell sheet onto the chondral defect is the only procedure in addition to routine surgery that participants will receive in this clinical study. Nonetheless, the safety of the cell sheet has been well demonstrated through tumorigenicity assessments conducted as part of validation testing. Thus, adverse events specific to this study are unlikely as the procedures involve the same level of risk as routine knee joint surgery performed under general anesthesia.

１１．Handling of adverse events

１）Symptoms or diseases

All undesired or unintended signs, symptoms, and diseases occurring postoperatively will be treated as adverse events. Exacerbation of the severity of complications will also be considered as an adverse event. Worsened degree of efficacy parameters will not be considered an adverse event.

２）Objective findings

Parameters compared to baseline values* (prior to start of the clinical study) that have become abnormal (normal → abnormal, abnormal → worse) by the day of the final examination will be considered adverse events. Furthermore, missing baseline values* that have become abnormal after the cell transplantation will be treated as adverse events. In the case of missing values, however, the value at 30 or more days before the date of obtaining consent will be considered the reference values.

*: Test values during the observation period following obtaining of consent (the results taken

closest to the initiation of treatment will be considered for tests conducted multiple times).

Data taken at onset, at maximum severity, at the time of assessment of outcome, and data considered critical for determining the relationship of the adverse event are to be noted on the patient's chart regardless of whether the adverse event has been defined in this study protocol.

３） Recording and investigation of adverse events

The signs and symptoms of an adverse event, details of objective findings, date, degree, severity, whether or not an action was taken and its details, outcomes and the day of determining the outcome of the adverse event, relationship with the clinical study, and rationale are to be noted on the patient's chart. If a disease name is to be noted, the symptoms associated with the disease are not noted as an adverse event.

Symptoms and diseases observed during the treatment period and objective findings that indicate an adverse event are generally to be followed up until the patient's condition has normalized or has recovered to a level that the condition is no longer considered an adverse event, regardless of the relationship of causality with this clinical study. However, this shall exclude cases in which the Principal Investigator physician or co-investigator physician has achieved a recovery, in which case the reasons for defining that the patient has recovered is to be noted on the charts. Irreversible adverse events such as organic disorders (brain or cardiac infarction) shall be followed up until the symptoms have stabilized or have stopped evolving.

４） Classification of adverse events

Adverse events are classified according to the following criteria:

① Mild: adverse events that do not affect the patient's daily living.

② Moderate: adverse events that affect the patient's daily living, but activities can be carried out with significant effort.

③ Severe: adverse events that cause major hindrance to the execution of the patient's ADL.

The outcomes of adverse events are classified according to the following criteria:

① Recovery: condition has normalized or has recovered to levels such that it is not considered an adverse event.

② Non-recovery: adverse events that have not recovered at that the assessed time point.

③ Unknown (death): outcome unknown due to the death of the patient.

５） Determining the association between adverse events and this clinical study

Associations of adverse events with this clinical study are determined according to the following criteria in consideration of the participant's condition, temporal relationship with the treatment, and

possible other causes:

① Clear association

② Probable association

③ Possible association

④ No association

Events determined to have levels of association ① to ③ with this clinical study are defined as adverse events for which an association with this clinical study cannot be excluded, and events with association ④ with this clinical study are determined as adverse events for which an association with this clinical study can be excluded.

６） Serious adverse events

The Principal Investigator physician or co-investigator physician promptly takes appropriate measures to treat patients with serious adverse events that occur during the treatment period regardless of the causal relationship with this clinical study. The Principal Investigator physician promptly reports the adverse event to the hospital Chairman and Chairman of the Ethics Review Board of the School of Medicine and discontinues the study even if 10 patients have not yet been enrolled in the clinical study.

【Serious adverse events】

1） Death

2） Life-threatening adverse event

3） Disability

4） Adverse events that may indirectly cause disability

5） Other adverse events of equal seriousness as 1) to 4)

6) Congenital anomaly or birth defects in children

７） Supplying new information

New information related to safety that is obtained through this clinical study shall be promptly reported in writing to the hospital Chairman, Dean of the School of Medicine, by the Principal Investigator physician and co-investigator physicians. The Principal Investigator physician and co-investigator physicians shall provide additional explanations to the participants, and make revisions to the information sheet and consent form as needed.

１２． Medical fees and indemnities

１）**Medical fees**

Costs associated with the cell sheet transplantation are covered entirely by the research funds of the Tokai University School of Medicine, and there are no costs to be paid out of pocket by the patient.

２）**Indemnities**

This study is covered by insurance for clinical trials; thus, any health injury that occurs in this clinical trial for which the indemnity liability arises can be compensated by what is covered by the policy.

１3. Statistical considerations

All comparable parameters will be compared to the greatest extent possible.

１4. Publication of study results

The results obtained in this study may be published in conferences or as an article. Personal information (privacy) of patients will be protected in publishing the results.

１５．Handling of test specimens after completion of the study

The cells obtained through this study are very valuable and can only be obtained through surgery; thus, any residual specimens (chondrocytes, synoviocytes) that remain after completion of the study may be used for other studies with the consent of the participant (see informed consent form) in linkable anonymity.

１６．References

1. Sato M, Mitani Satoshi Ito G, Ukai T, Kokubo M, JMochida J: Cartilage cell sheet for articular cartilage defect repair, diseases in orthopaedic surgery observed from the molecular level－Series VIII. Orthopaedic and Disaster Surgery, 53(13), 2010.
2. Sato M, Cell sheet technologies for cartilage repair, Regenerative medicine and biomaterials for the repair of connective tissues, WOODHEAD Publishing, CBC Press LLC, 251-265,

2010.

3. Kaneshiro N, Sato M, Ishihara M, Mitani G, Sakai H, Mochida J: Bioengineered chondrocyte sheets may be potentially useful for the treatment of partial thickness defects of articular cartilage. Biochem Biophys Res Commun 349 (2), 723-731, 2006.
4. Mitani G, Sato M, Lee J IK, Kaneshiro N, Ishihara M, Ota N, Kokubo M, Sakai H, Kikuchi T, Mochida J: The properties of bioengineered chondrocyte sheets for cartilage regeneration. BMC Biotechnol. 9(17), 2009.
5. Kaneshiro N, Sato M, Ishihara M, Mitani G, Sakai H, Kikuchi T, Mochida J: Cultured articular chondrocytes sheets for partial thickness cartilage defects utilizing temperature・responsive culture dishes. Eur Cell Mater 13, 87-92, 2007.
